# Butyrate promotes oral squamous cell carcinoma cells migration, invasion and epithelial-mesenchymal transition

**DOI:** 10.7717/peerj.12991

**Published:** 2022-02-22

**Authors:** Wenli Zang, Junchao Liu, Fengxue Geng, Dongjuan Liu, Shuwei Zhang, Yuchao Li, Yaping Pan

**Affiliations:** 1 Department of Periodontology, School and Hospital of Stomatology, China Medical University, Shenyang, China; 2 Department of Emergency and Oral Medicine, School and Hospital of Stomatology, China Medical University, Shenyang, China

**Keywords:** Butyrate, Migration, Invasion, Epithelial-mesenchymal transition, Oral squamous cell carcinoma

## Abstract

**Background:**

Oral squamous cell carcinoma (OSCC), the most common type of primary malignant tumor in the oral cavity, is a lethal disease with high recurrence and mortality rates. Butyrate, a metabolite produced by periodontal pathogens, has been linked to oral diseases. The purpose of this study was to evaluate the effect of sodium butyrate (NaB) on the proliferation, migration, and invasion of OSCC cells *in vitro* and to explore the potential mechanism.

**Methods:**

Two OSCC cell lines (HSC-4 and SCC-9) were treated with NaB at different concentrations. The cell proliferation was assayed by CCK-8, ethylene deoxyuridine (EdU), and flow cytometry. Wound healing and transwell assay were performed to detect cell migration and invasion. Changes in epithelial-mesenchymal transition (EMT) markers, including E-cadherin, Vimentin, and SNAI1, were evaluated by quantitative real-time PCR (qRT-PCR), western blot, and immunofluorescent staining. The expression levels of matrix metalloproteinases (MMPs) were analyzed by qRT-PCR and gelatin zymography.

**Results:**

Our results showed that NaB inhibited the proliferation of OSCC cells and induced cell cycle arrest at G1 phase, but NaB significantly enhanced cell migration and invasion compared with the control group. Further mechanistic investigation demonstrated that NaB induced EMT by increasing the expression of Vimentin and SNAI1, decreasing the expression of membrane-bound E-cadherin, and correspondingly promoting E-cadherin translocation from the membrane to the cytoplasm. In addition, the overexpression of MMP1/2/9/13 was closely related to NaB treatment.

**Conclusions:**

Our study conclude that butyrate may promote the migration and invasion of OSCC cells by inducing EMT. These findings indicate that butyrate may contribute to OSCC metastasis.

## Introduction

Oral cancer is one of the most common malignant tumors, with 377,713 new cases and 177,757 deaths in 2020 worldwide (Sung et al., 2021). Oral squamous cell carcinoma (OSCC) is the most common histological type of oral cancer (Leemans, Braakhuis & Brakenhoff, 2011). The high incidence of local lymph node metastasis and the invasion of distant organs are the major causes of OSCC-related death (Bhattacharya et al., 2011). Despite significant advances in therapeutic intervention and prevention of OSCC, the prognosis is still poor. Therefore, more efforts should be directed to reveal the pathogenesis and development of OSCC.

The causes of OSCC are multifactorial, apart from tobacco and alcohol consumption, infection by human papilloma viruses (HPVs), and betel nut chewing, which have been thoroughly studied, bacterial infection and chronic inflammation are increasingly considered important determinants for the development of OSCC (Hernandez et al., 2017; Hübbers & Akgül, 2015; Nasry, Rodriguez-Lecompte & Martin, 2018). Previous studies have confirmed that higher counts of periodontal pathogens such as *Porphyromonas gingivalis* and *Fusobacterium nucleatum* are present in OSCC tissues compared with the corresponding normal tissues, and these pathogens might aggravate immune responses and chronic inflammation so as to promote carcinogenesis and cancer development (Li et al., 2018; Plottel & Blaser, 2011). Furthermore, several reports have suggested that metabolites of anaerobic bacteria are also highly related to cancer progression. Short-chain fatty acids (SCFAs), such as acetate and butyrate, are important virulence factors produced by periodontal pathogens through anaerobic glycolysis. Among the SCFAs, butyrate has been demonstrated to contribute to the occurrence and development of periodontitis (Cueno & Ochiai, 2015). Moreover, a previous study by Ohshima et al. (2017) showed higher concentration of butyrate in saliva obtained from patients with OSCC compared with that from healthy controls, which could be regarded as one of the potential biomarkers of OSCC. In addition, a metabolomics analysis on OSCC tissues performed by Tsai et al. (2020) indicated that higher levels of butyrate in tumor tissues were associated with increased tumor stage and extranodal extension of metastatic nodes. Nevertheless, the effect of butyrate on OSCC cell metastasis remains unclear, and little is known about the potential underlying mechanism. Therefore, further investigation of the role of butyrate in the progression of OSCC is warranted.

Epithelial-mesenchymal transition (EMT) is a critical step in the progression, invasion, and metastasis of tumor cells, as it involves conversion of epithelial cells into motile mesenchymal cells (Chaffer et al., 2016). The loss of cell-cell adhesions and the acquisition of mesenchymal characteristics, such as downregulation of E-cadherin and upregulation of Vimentin, endow tumor cells with migratory and invasive properties (Heerboth et al., 2015). This switch in cell behavior is controlled by a group of transcription factors including snail family transcriptional repressor 1 (SNAI1), snail family transcriptional repressor 2 (SLUG), zinc finger E-Box binding homeobox (ZEB), and twist basic helix-loop-helix transcription factor (TWIST) (Simeone et al., 2019). SNAI1 is a master EMT regulator, which can repress the transcription of epithelial-specific genes such as E-cadherin and upregulate mesenchymal genes such as Vimentin (Barrallo-Gimeno & Nieto, 2005). It has been shown that SNAI1-associated EMT occurs in OSCC and potentially facilitates tumor progression (Schwock et al., 2010). Importantly, cells undergoing EMT also regulate the expression of matrix metalloproteinases (MMPs), which are well-known proteolytic enzymes that can degrade extracellular matrix (ECM) and are associated with cancer progression (Gialeli, Theocharis & Karamanos, 2011). In addition, some studies have demonstrated that butyrate, acting as a histone deacetylase inhibitor, could inhibit EMT by regulating E-cadherin expression and subcellular distribution, while other studies suggested that butyrate could induce EMT (Serpa et al., 2010; Huang, Wu & Wang, 2019).

Therefore, the purpose of this study was to ascertain the effect of butyrate on OSCC cell migration and invasion and to elucidate the underlying mechanism. We showed that butyrate at clinically relevant concentrations (as observed during periodontitis) (Niederman et al., 1997) significantly promoted OSCC cell migration and invasion by inducing EMT, as evidenced by the upregulation of SNAI1, Vimentin, and MMP1/2/9/13 and the functional loss of E-cadherin. These results suggest that butyrate can drive the progression of OSCC.

## Materials and methods

### Cell lines and cell culture

HSC-4 and SCC-9 cells, immortalized human OSCC cell lines, were obtained from the Japanese Collection of Research Bioresources Cell Bank (JCRB, Shinjuku, Japan) and the American Type Culture Collection (ATCC, Manassas, VA, USA), respectively. HSC-4 cells were routinely cultured and passaged in DMEM/high glucose (Gibco, CA, USA) supplemented with 10% fetal bovine serum (FBS). SCC-9 cells were cultured and passaged in DMEM/Ham’s F-12 medium with 10% FBS. The two human OSCC cell lines were both incubated at 37 °C and 5% CO_2_ and treated with sodium butyrate (NaB) (Sigma-Aldrich,St. Louis, MI, USA) at the indicated concentrations.

### Cell viability assay

The CCK-8 assay was used to detect cell viability as previously described (Qiu et al., 2017). Briefly, HSC-4 and SCC-9 cells were inoculated into 96-well plates at a density of 3 × 10^3^ cells per well and treated with NaB at different concentrations (0, 1.25, 2.5, and 5 mM). After incubation for 24 and 48 h, 10 μl CCK-8 reagent (Beyotime Institute of Biotechnology, Shanghai, China) was added to each well, and the samples were incubated at 37 °C in the dark for another 2 h. The absorbance at 450 nm was determined by a microplate reader (Tecan, Mechelen, Belgium). All experiments were performed in three biological replicates.

### kFluor488 Click-iT EdU assay

EdU detection kit (5-Ethynyl-2′-deoxyuridine) (KeyGENBioTECH, Nanjing, China) was used to assess cell proliferation in line with the producer’s instructions. A total of 5 × 10^4^ OSCC cells per well were seeded on 24-well plates. After treatment with different concentrations of NaB (0, 2.5, or 5 mM) for 24 h, 10 μM EdU labeling media was added to the culture cells for another 2 h, and then the cells were stained with 1× Click-iT EdU staining mix for 30 min in the dark. These cells were subsequently counterstained with Hoechst 33342 for 20 min and imaged by a fluorescence microscope (200×) (Nikon DS-Ri2).

### Cell cycle analysis

OSCC cells (3 × 10^5^) were seeded in six-well plates and treated with different concentrations of NaB (0, 2.5 or 5 mM) for 24 h. For cell cycle analysis, Cell Cycle and Apoptosis Analysis Kit (Beyotime Biotech. Co., Shanghai, China) was used. Briefly, the cells were digested and fixed in 70% ethanol at 4 °C overnight. Next, they were collected and washed with cold PBS and then incubated with 0.5 ml propidium iodide (PI) staining buffer in the dark for 30 min. The cell cycle was assessed using flow cytometry (FACS; BD, Franklin Lake, NJ, USA).

### Wound healing assay

To assess cell migration, HSC-4 and SCC-9 cells were seeded at 4 × 10^5^ cells per well in six-well plates in medium with 10% FBS at 37 °C and 5% CO_2_. After reaching approximately 90% confluence, the cells were wounded by scraping with a 200 μl pipette tip two times per well, followed by three careful washes with PBS, and subsequently incubated in serum-free medium containing different concentrations of NaB (0, 2.5, or 5 mM). Then wounds were observed and photographed at eight sites by microscopy with 100× magnification at 0 h and 24 h. The wound area was determined by ImageJ software.

### Transwell assay

HSC-4 and SCC-9 cell migration was further analyzed by 24-well Transwell chambers (pore size, 8 μm; Corning, NY, USA). HSC-4 and SCC-9 cells were seeded into the upper chambers (1 × 10^5^ cells per well) in 200 μl serum-free medium and treated with NaB (0, 2.5, or 5 mM); 600 μl of medium with 10% FBS was added to the lower chambers. After incubation for 24 h at 37 °C, the cells that had migrated through the membrane were fixed in 4% paraformaldehyde for 30 min and stained with 0.2% crystal violet dye for 20 min. Then, the chambers were washed with distilled water and photographed under the microscope, and cells were counted. The cells that had traversed the membrane were counted in five different fields (200×). For transwell invasion assay, Matrigel (BD Biosciences, Franklin Lakes, NY, USA) was used to precoat the upper chamber of transwell for 1 h at 37 °C, and the cells were incubated for 48 h.

### Quantitative real-time PCR

HSC-4 and SCC-9 cells (2.5 × 10^5^ cells per well) were plated in six-well plates in medium containing 10% FBS and NaB (0, 2.5 or 5 mM) at 37 °C and 5% CO_2_. Total cellular RNA from OSCC cells was extracted with TRIzol reagent (ThermoFisher Scientific, Waltham, MA, USA) and cDNA was synthesized using the PrimeScript™ RT Reagent Kit (Takara Bio Inc., Dalian, China). Then, quantitative real-time PCR (qRT-PCR) was performed with a 7,500 Real-time PCR system using the TB Green Premix Ex Taq II kit (Takara Bio Inc., Dalian, China). The primers used in this study are listed in Table 1. Each sample was analyzed in triplicate and the relative expression was analyzed using the comparative 2^−ΔΔCt^ determination method.

**Table 1:** Primers used for qRT-PCR.

Gene	Forward primer 5′-3′	Reverse primer 5′-3′
MMP1	CCAGATGTGGAGTGCCTGATGTG	CTCAGAGACCTTGGTGAATGTCAGAG
MMP2	CACCTACACCAAGAACTTCCGTCTG	GTGCCAAGGTCAATGTCAGGAGAG
MMP9	TGTACCGCTATGGTTACACTCG	GGCAGGGACAGTTGCTTCT
MMP13	CCTGGCTGCCTTCCTCTTCTTG	GCCTCTCAGTCATGGAGCTTGC
N-cadherin	AACAGCAACGACGGGTTAGT	CAGACACGGTTGCAGTTGAC
SNAI1	TCGGAAGCCTAACTACAGCGA	AGATGAGCATTGGCAGCGAG
Vimentin	AGGCGAGGAGAGCAGGATTT	AGTGGGTATCAACCAGAGGGA
E-cadherin	CCTGGGACTCCACCTACAGAA	AGGAGTTGGGAAATGTGAGC
Twist	GTCCGCAGTCTTACGAGGAG	GCTTGAGGGTCTGAATCGGGCT
SLUG	GCTACCCAATGGCCTCTCTC	CTTCAATGGCATGGGGGTCT
GAPDH	GAAGGTGAAGGTCGGAGTC	GAAGATGGTGATGGGATTTC

### Western blot analysis

HSC-4 and SCC-9 cells (1.25 × 10^6^ cells per dish) were incubated in 100-mm Petri dishes in a medium with indicated concentrations of NaB (0, 2.5, or 5 mM) for 24 h. Then, the cells were collected and lysed in RIPA buffer (Beyotime Biotechnology, Shanghai, China) containing 1:100 protease inhibitors phenylmethanesulfonyl fluoride (Beyotime, Shanghai, China) for 40 min to extract the total protein fraction. In order to separate membrane-bound and cytosolic proteins, we used a membrane and cytosolic protein extraction kit (Beyotime Biotechnology, Shanghai, China) according to the manufacturer’s instructions. The protein concentrations were determined by the BCA protein assay kit (Beyotime Biotechnology, Shanghai, China). Proteins (30 μg per lane) were separated by 8% SDS-polyacrylamide gel electrophoresis and transferred onto nitrocellulose membranes (Pall, NY, USA). After blocking by 5% skim milk in TBST for 1.5 h, the membranes were incubated with anti-β-actin (1:1,000; Abbkine, Beijing, China), anti-SNAI1 (1:1,000; Cell Signaling Technology Inc., Boston, MA, USA), anti-E-cadherin (1:3,000; Abcam, Cambridge, MA, USA), and anti-Vimentin (1:500; Abcam, Cambridge, MA, USA) overnight at 4 °C. Then, the membranes were washed and incubated with Dylight-800-conjugated goat anti-rabbit/anti-mouse IgG secondary antibodies (1:2,000; Abbkine, Beijing, China). After additional washing steps, protein bands were visualized using Odyssey CLX (LI-COR, Lincoln, NE, USA) and signals were quantified using ImageJ software.

### Immunofluorescent staining

HSC-4 and SCC-9 cells (5 × 10^4^ cells per well) were seeded on 24-well plates and treated with NaB (2.5 or 5 mM) for 24 h. Then, the treated cells were fixed with 4% paraformaldehyde for 30 min at 4 °C. After that, cells were washed with PBS, permeabilized for 5 min in PBS containing 0.5% Triton X-100, and blocked for 1 h with PBS containing 1% BSA at room temperature. Subsequently, primary antibodies against E-cadherin, Vimentin, and SNAI1 (1:200 in PBS containing 1% BSA) were incubated overnight at 4 °C. After washing with PBS, cells were immunostained with Alexa Fluor 594-conjugated secondary antibodies (Proteintech Group, Chicago, IL, USA) at 1:200 dilution protected from light at room temperature for 1 h. Finally, the immunostained cells were counterstained with DAPI (Beyotime Biotech. Co., Shanghai, China) and images were captured with a fluorescence microscope (Nikon DS-Ri2).

### Gelatin zymography

To evaluate the levels of protein secretion of MMP2 and MMP9 by HSC-4 and SCC-9 cells, zymography assay was performed using the MMP Zymography Assay Kit (Applygen Technologies Inc., Beijing, China) in accordance with the manufacturer’s instructions. HSC-4 and SCC-9 cells were incubated in the serum-free medium supplemented with NaB (2.5 or 5 mM) for 24 h. Then, the supernatant was collected and protein concentrations were measured by BCA protein assay kit (Beyotime Biotechnology, Shanghai, China). Total protein (150 μg) was separated by electrophoresis in 8% SDS-polyacrylamide gels containing 0.1% gelatin substrate. The gels were incubated at room temperature with Buffer A for 4 h on a shaker. Subsequently, the gels were incubated in Buffer B for 48 h at 37 °C. Then, the gels were stained with Coomassie brilliant blue R-250 to visualize active bands and de-stained with 30% methanol and 10% acetic acid solution. Densitometry was performed using ImageJ software.

### Statistical analysis

All experiments were repeated three times. Statistical analyses were performed by GraphPad Prism 7.0, and one-way analysis of variance followed by Dunnett’s multiple-comparison tests was applied to compare multiple groups. All comparisons were made with respect to the untreated groups. A *P*-value < 0.05 was considered statistically significant.

## Results

### Butyrate inhibits OSCC cells proliferation and induces cell cycle arrest *in vitro*

**Figure 1:**
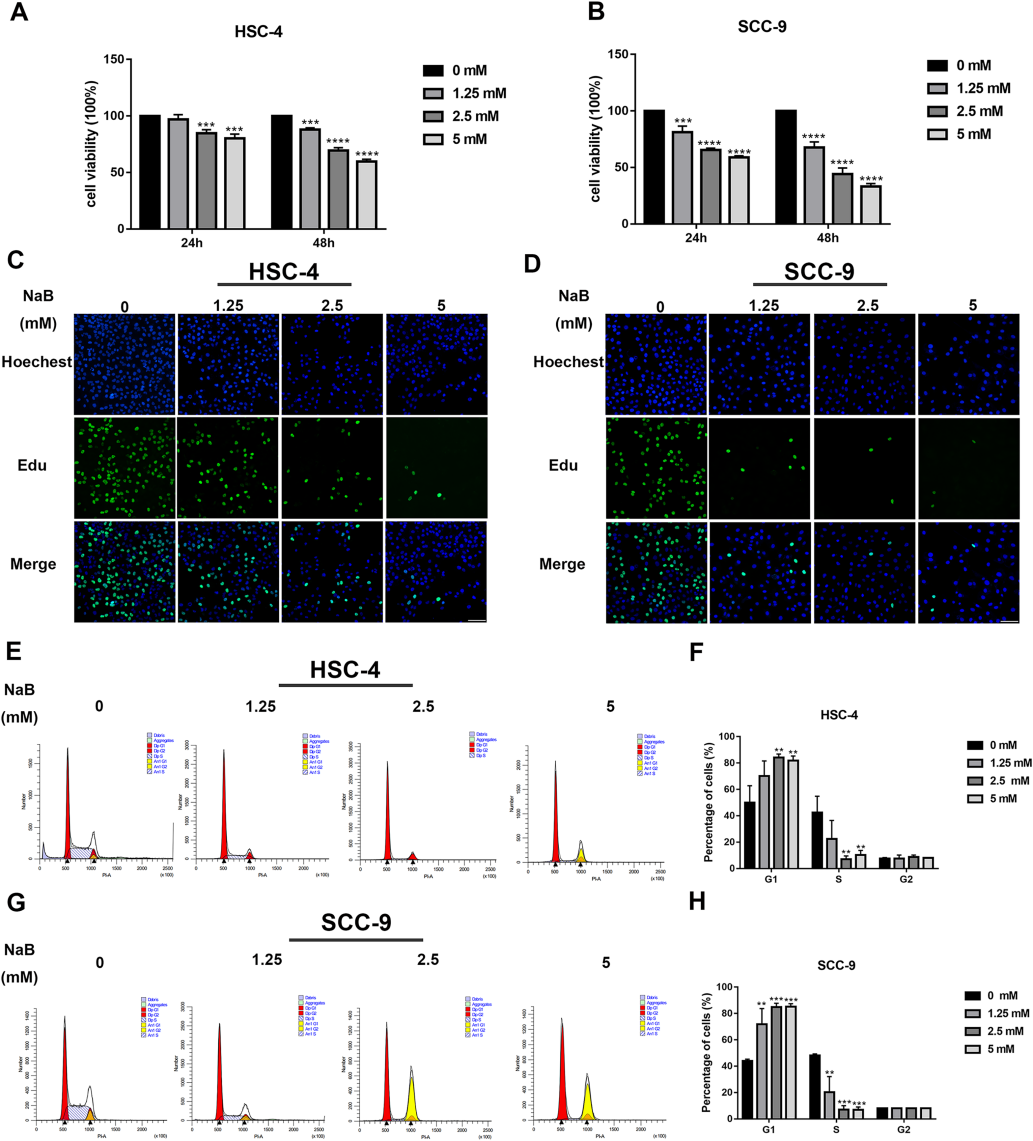
NaB suppresses OSCC cells’ proliferation and induces cell cycle arrest at G1 phase. (A & B) The viability of HSC-4 and SCC-9 cells treated with NaB (0, 1.25, 2.5, or 5 mM) was examined by CCK-8 assay. NaB significantly decreased cell viability in a concentration- and time-dependent manner. (C & D) The effect of 24-h NaB treatment on the proliferation of HSC-4 and SCC-9 cells was further detected by EdU incorporation assay. Scale bar = 100 μm. (E)–(H) The cell cycle distribution of HSC-4 and SCC-9 cells treated with NaB was investigated by flow cytometry assay. Data are presented as the mean ± SD of at least three independent experiments. NaB, sodium butyrate. **P < 0.01, ***P < 0.001, ****P < 0.0001 *vs* control (NaB, 0 mM).

The viability of OSCC cells treated with NaB (0, 1.25, 2.5, or 5 mM) *in vitro* was assayed by CCK-8. Our data showed that concentrations of NaB used in this study were able to significantly inhibit HSC-4 and SCC-9 cell viability in a concentration- and time-dependent manner (Figs. 1A and 1B). In particular, NaB treatment for 48 h inhibited cell viability to approximately 50% in SCC-9 cells. Subsequently, to further evaluate the effects of NaB on cell proliferation in OSCC cells, EdU assay was carried out. As shown in Figs. 1C and 1D, the proliferation of HSC-4 and SCC-9 cells was obviously inhibited after treatment with NaB for 24 h. The cell cycle of OSCC cells was also assessed by Cell cycle analysis. We observed that NaB increased the percentage of G1 phase cells and decreased the percentage of S phase cells (Figs. 1E–1H). These results indicated that NaB induced cell cycle arrest at the G1 phase in OSCC cells.

### Butyrate promotes OSCC cell migration and invasion

**Figure 2:**
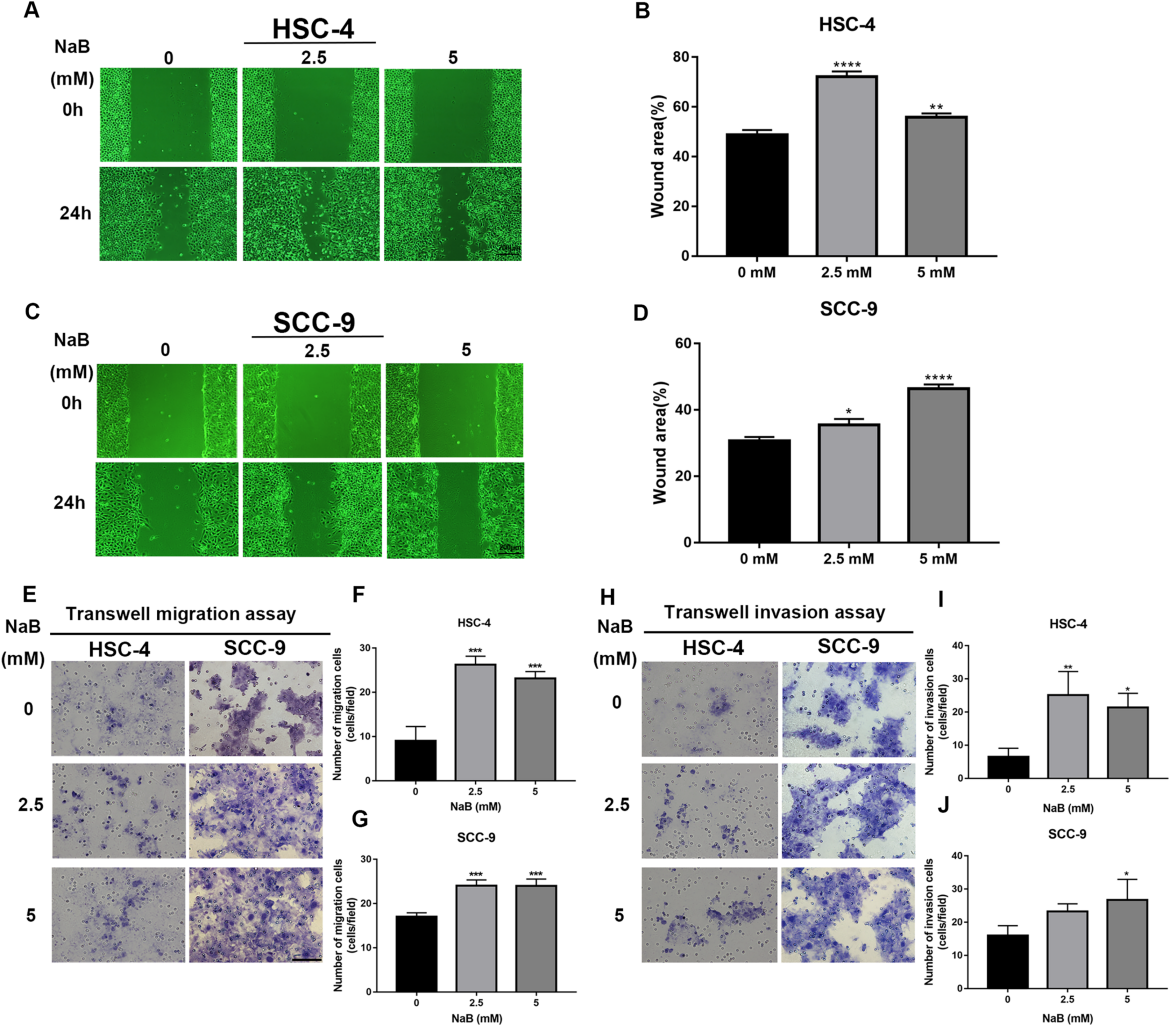
The promoting effect of NaB on OSCC cells migration and invasion. (A–D) Wound healing assay was used to detect the horizontal mobility of HSC-4 and SCC-9 cells after treatment with NaB (0, 2.5, or 5 mM) for 0 and 24 h. Scale bar represents 200 μm. (E–G) Furthermore, the migration ability of HSC-4 and SCC-9 cells after treatment with NaB (0, 2.5, or 5 mM) for 24 h was tested by transwell migration assay. Scale bar represents 100 μm. (H–J) Transwell invasion assay of HSC-4 and SCC-9 cells after treatment with NaB (0, 2.5, or 5 mM) for 24 h. Scale bar represents 100 μm. Data are presented as the mean ± SD of at least three independent experiments. NaB, sodium butyrate. *P < 0.05, **P < 0.01, *** *P* < 0.001, ****P < 0.0001 *vs* control (NaB, 0 mM).

The wound healing assay and transwell assay were used to investigate whether NaB can affect the migration and invasion abilities of HSC-4 and SCC-9 cells. Our data showed that the cell migration ability significantly increased after NaB treatment for 24 h (Figs. 2A–2D). Moreover, NaB treatment at 2.5 mM promoted HSC-4 cell migration better than other concentrations, while NaB stimulated cell migration in a dose-dependent manner in SCC-9 cells. Similarly, Matrigel invasion assay showed that NaB increased the invasion ability of the two OSCC cell lines compared with the control group, which was consistent with the results of the migration assay (Figs. 2E–2J). These findings suggested that NaB stimulation enhanced OSCC cells’ migration and invasion abilities.

### Butyrate induces EMT in OSCC cells

**Figure 3:**
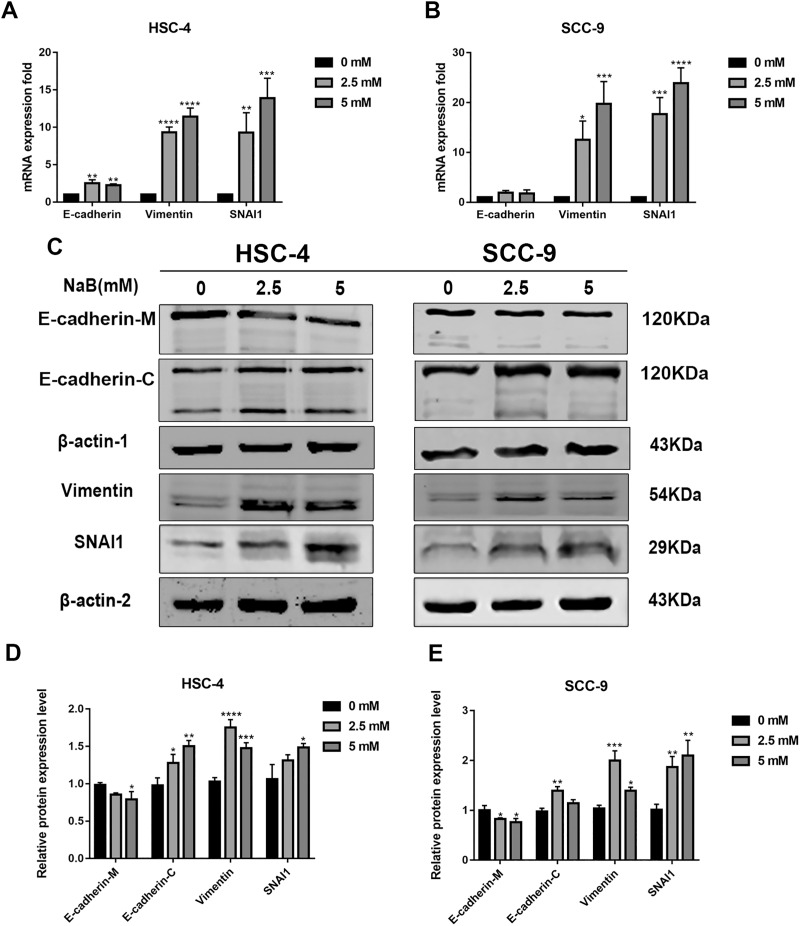
NaB promotes the expression of mesenchymal markers and increases internalization of E-cadherin into the cytosol. (A & B) Gene expression of E-cadherin, Vimentin and SNAI1 in HSC-4 and SCC-9 cells after treatment with NaB for 24 h was detected by qRT-PCR analysis. (C–E) NaB promotes the expression of SNAI1 and Vimentin, reduces membrane-bound E-cadherin and correspondingly increases cytosolic E-cadherin in HSC-4 and SCC-9 cells. β-actin was used as a housekeeping gene. Data are presented as the mean ± SD of at least three independent experiments. *P < 0.05, **P < 0.01, ***P < 0.001, ****P < 0.0001 *vs* control (NaB, 0 mM).

**Figure 4:**
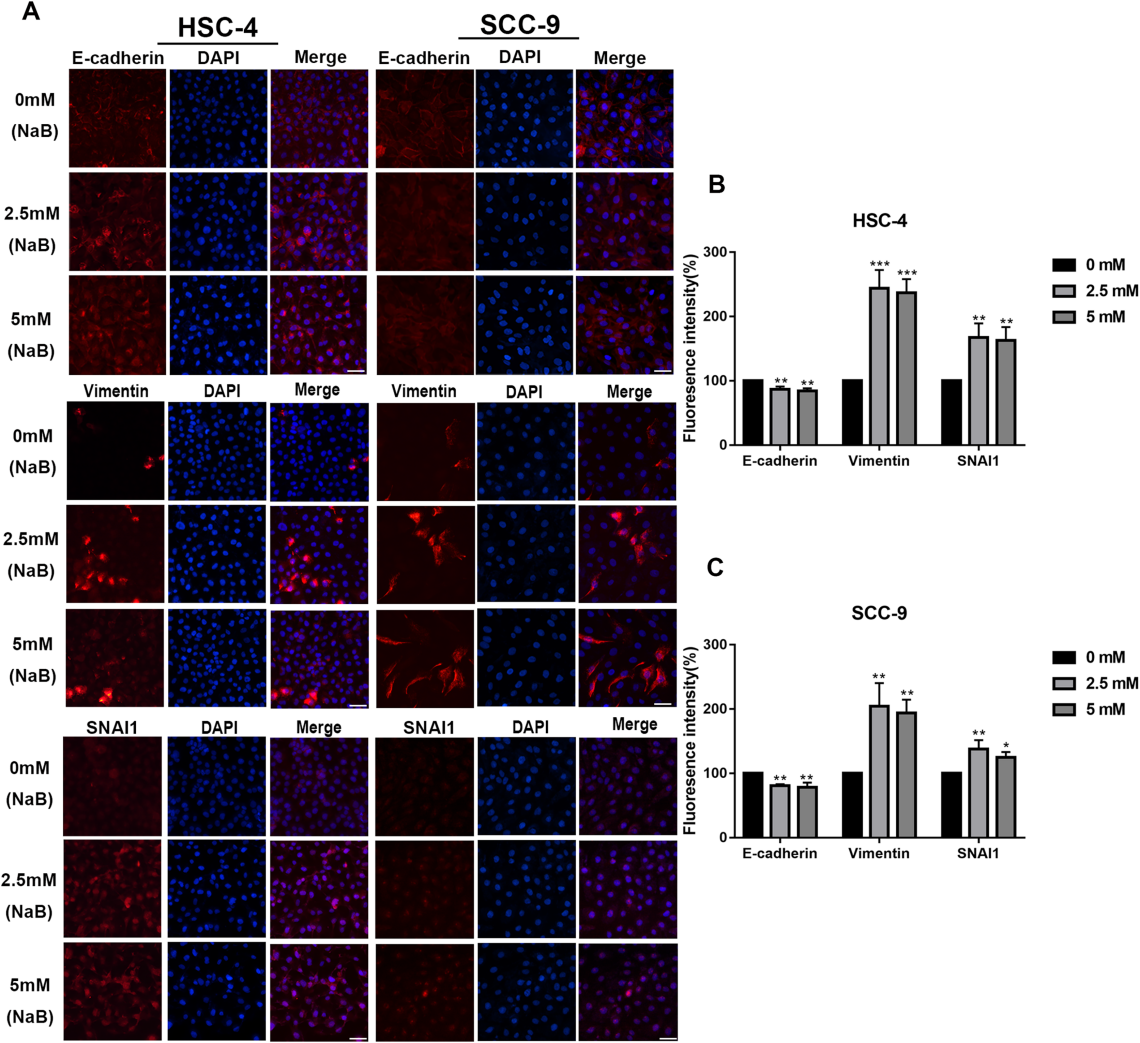
Immunofluorescence of mesenchymal markers. (A–C) Representative images of the expression and localization of E-cadherin, Vimentin and SNAI1 visualized by immunofluorescence in HSC-4 and SCC-9 cells treated with NaB (0, 2.5, or 5 mM). Scale bar represents 50 µm. Data are presented as the mean ± SD of at least three independent experiments. *P < 0.05, **P < 0.01, ***P < 0.001 *vs* control (NaB, 0 mM).

EMT has been linked to tumor cell migration and invasion in various cancers, including OSCC. To explore the impact of NaB on EMT in OSCC cells, we evaluated the expression and localization of EMT-associated markers such as SNAI1, Vimentin, and E-cadherin. As shown in Figs. 3A and 3B, NaB remarkably increased the mRNA expression levels of Vimentin and SNAI1 and slightly increased the mRNA expression of E-cadherin. Increases in protein expression levels of SNAI1 and Vimentin were also observed by western blot analysis (Figs. 3C–3E). Furthermore, the impact of NaB on the expression levels of SLUG, Twist, and N-cadherin was also detected by qRT-PCR (Fig. S1). The results showed that NaB slightly increased the mRNA expression of SLUG and N-cadherin compared with the untreated controls. E-cadherin is a transmembrane glycoprotein that is involved in cell-cell adhesion, and when it is internalized into the cell, its function changes (Du et al., 2014). Therefore, in order to accurately examine the changes of E-cadherin expression, we separately measured membrane-bound and cytosolic protein levels. As shown in Figs. 3C–3E, the cytosolic protein expression of E-cadherin was enhanced, whereas the membrane-bound protein expression was significantly decreased after treatment with NaB for 24 h. Similar results were obtained by immunofluorescence staining (Figs. 4A–4C). The immunofluorescence results showed that NaB changed the location of E-cadherin; namely, E-cadherin was translocated from the membrane to the cytoplasm in NaB-treated cells, especially in HSC-4 cells, which further proved that cell adhesion was disrupted and cell architecture was altered, indicating EMT.

### Butyrate upregulates the expression of MMPs in OSCC cells

**Figure 5:**
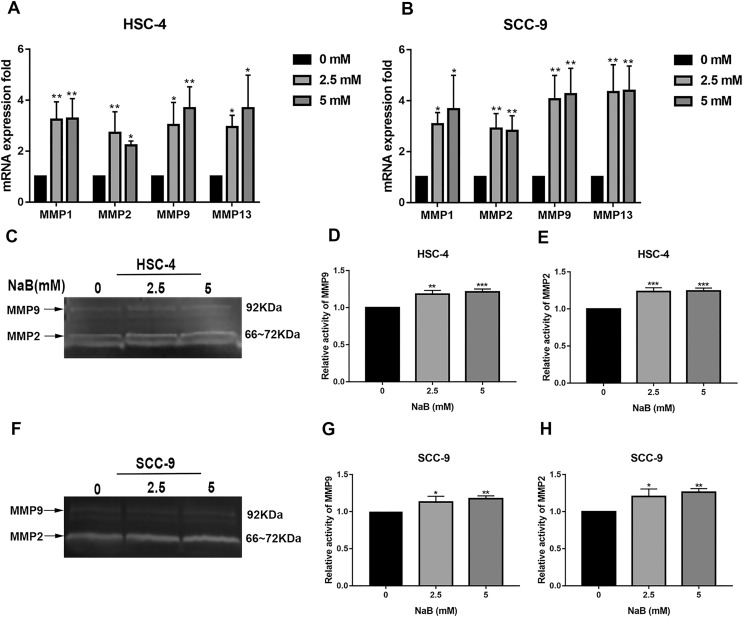
Effects of NaB on the expression of MMPs. (A & B) MMPs mRNA was detected by qRT-PCR using GAPDH as a control in HSC-4 and SCC-9 cells treated with NaB (0, 2.5, or 5 mM). (C–H) The amount of MMP2 and MMP9 was measured by gelatin zymography. Data are presented as the mean ± SD of at least three independent experiments. *P < 0.05, **P < 0.01, ***P < 0.001 *vs* control (NaB, 0 mM).

It has been shown that MMPs play crucial roles in tumor progression and metastasis by cleaving ECM (Rydlova et al., 2008). As previously reported, the expression of MMPs is associated with SNAI1 expression and is usually upregulated during EMT (Krisanaprakornkit & Iamaroon, 2012). Therefore, to elucidate the mechanism by which NaB stimulates cell migration, we evaluated the expression of MMP1, MMP2, MMP9, and MMP13, which are related to cell migration and invasion, by qRT-PCR. As shown in Figs. 5A and 5B, the MMP1, MMP2, MMP9, and MMP13 mRNA levels in HSC-4 and SCC-9 cells increased two- to seven-fold after NaB treatment for 24 h. Among the MMPs, MMP2 and MMP9 are activated upon secretion and are considered to be the most relevant to cancer migration and invasion (Deryugina & Quigley, 2006). Therefore, to further investigate whether the OSCC cells treated with NaB for 24 h produce more active forms of MMP2 and MMP9, the protein levels of MMP2 and MMP9 were examined by gelatin zymography. The results showed slightly increased secretion of MMP2 and MMP9 in the two cell lines treated with NaB.

## Discussion

OSCC normally involves the tongue, lips, and floor of mouth, adversely affecting the patient’s appearance and quality of life. It is characterized by highly invasive mode and is often diagnosed at advanced stages, leading to a low 5-year survival rate (Gigliotti, Madathil & Makhoul, 2019). Accumulating evidence indicates that the incidence of OSCC has increased in recent years (Ren et al., 2020). The search for effective diagnostic biomarkers and the underlying mechanisms is therefore urgent. Butyrate, one of the typical SCFAs, is usually produced during periodontal pathogens fermentation in the oral cavity. As a histone deacetylase inhibitor, butyrate has been considered to play a significant role in the metastasis of multiple tumor cells (Xiao, Xu & Chen, 2019; Gonçalves & Martel, 2013), but its function in OSCC is still unclear.

In the present study, we performed a series of experiments to examine the effects of butyrate on OSCC cells. Our results demonstrated that butyrate significantly inhibited OSCC cells’ proliferation and induced cell cycle arrest at the G1 phase. In addition, we surprisingly observed that butyrate promoted cell migration and invasion, which was also explored in previous studies (Miyazaki et al., 2015; Xu et al., 2018). In this study, we found that the effect of butyrate on motility of OSCC cells was not consistent with the effect on colorectal cancer and ovarian cancer (Zuo et al., 2013; Mrkvicova et al., 2019). In oral cavity, butyrate is a metabolite of anaerobic bacteria such as *P. gingivalis*, *Prevotella intermedia*, and *F. nucleatum* (Lu et al., 2014). Our previous studies have confirmed that both *P. gingivalis* and *F. nucleatum* can promote the occurrence and development of OSCC (Zhang et al., 2020; Chang et al., 2019). Therefore, we speculate that butyrate may have different effects on cancer cells from different tissues.

Based on this finding, we further investigated the expression of EMT-associated molecular markers. EMT has been reported to be a crucial step in promoting tumor cell migration *via* reducing cell-cell adhesion and actin cytoskeleton reorganization (Miki et al., 2007). Previous studies have demonstrated that butyrate could influence the EMT process, but the exact effects remain controversial (Shin et al., 2016; Mrkvicova et al., 2019). E-cadherin is a critical epithelial marker to maintain cell-cell adhesion, and the loss of E-cadherin expression is considered a key event during the process of tumor metastasis (Chen et al., 2020). Another study has shown that the reduction of E-cadherin levels is associated with the upregulation of the mesenchymal marker Vimentin in OSCC progression, which indicates that cells have acquired the EMT phenotype (Beavon, 2000). In addition, SNAI1 is considered one of the most prominent suppressors of E-cadherin transcription (Chaw et al., 2012) and plays a key role in the development and progression of various kinds of cancers (Tsai & Yang, 2013; Bai et al., 2015). Another study has shown that SNAI1 is closely related to the modulation of migration and invasion of OSCC cells (Zhu et al., 2012). As anticipated, we found that butyrate significantly increased the expression of SNAI1 and Vimentin and slightly increased the expression of SLUG and N-cadherin in both HSC-4 cells and SCC-9 cells. Interestingly, we also observed an increase in cytosolic protein level of E-cadherin upon treatment with butyrate, while the membrane-bound protein level of E-cadherin decreased significantly, which proved that E-cadherin had been internalized to the cytosol from the cell membrane. Similarly, previous studies have indicated the abnormal expression of cytoplasmic E-cadherin in various cancer tissues, which was strongly associated with more aggressive tumor-related variables, such as poor grade levels and lymph node metastasis of patients (Ramesh, Nash & McCulloch, 1999; Bendardaf et al., 2019; Gao et al., 2005). It is therefore suggested that the abnormal localization of E-cadherin seem to be a marker of cancer progression.

MMPs are major hydrolytic enzymes that degrade ECM by proteolytic digestion during metastasis, and there is a clear connection between MMPs, ECM degradation, and cancer cell metastasis (Kaji et al., 2019). MMPs play significant roles in cell proliferation, migration, angiogenesis, and many other essential processes (Cui, Hu & Khalil, 2017). In addition, previous studies have shown that MMPs can also induce and facilitate EMT process of tumors (Radisky & Radisky, 2010). Moreover, it has been reported that SNAI1, a transcription factor, could also enhance the expression of MMPs in various invasive cancers (Ou, Guan & Yang, 2019; Singh, Deshmukh & Das, 2021). Our findings demonstrated that butyrate significantly upregulated the gene expression levels of MMP1, MMP2, MMP9, and MMP13 and stimulated the secretion of MMP2 and MMP9, which was consistent with SNAI1 fluctuations. Nevertheless, the molecular mechanisms underlying their upregulation by SNAI1 remain unclear (Jin et al., 2010). This issue will be the subject of further work. Collectively, our results highlight the role of butyrate in promoting the migration and invasion of OSCC cells, which could contribute to the elucidation of the mechanisms underlying OSCC and the development of novel therapeutics. Moreover, our results support a mechanism of butyrate-induced OSCC cell migration and invasion that involves the induction of EMT. This hypothesis needs to be further investigated in future studies. The lack of *in vivo* experiments is the deficiency of our study. We will carry out in-depth research on butyrate in the future, and *in vivo* experiments will be a part of our follow-up experiments.

In summary, our study demonstrates that butyrate treatment significantly contributes to OSCC cell migration and invasion, which is closely associated with the induction of EMT. Thus, butyrate may provide new insights into the mechanisms underlying metastasis of OSCC and provide a link between periodontitis and OSCC.

## Supplemental Information

10.7717/peerj.12991/supp-1 Supplemental Information 1 Raw data.

10.7717/peerj.12991/supp-2 Supplemental Information 2 Supplementary Figure.

## Additional Information and Declarations

### Data Availability

The following information was supplied regarding data availability: The raw measurements are available in the Supplemental File.
