# Supplementary material for: Butyrate promotes oral squamous cell carcinoma cells migration, invasion and epithelial-mesenchymal transition
Source: PeerJ. 2022 Feb 22;10:e12991. doi: 10.7717/peerj.12991 (PMC8877342; doi:10.7717/peerj.12991)
Supplement: Supplemental Information 1 [file peerj-10-12991-s001.zip › Raw data/Certificate of English Language Editing.pdf]

Certificate of English Language Editing

Manuscript Title:

Butyrate promotes migration, invasion, and epithelial–mesenchymal transition of oral squamous cell carcinoma cells

Date of Revision:

January 25, 2022

Abstract:

Background: Oral squamous cell carcinoma (OSCC), the most common type of primary malignant tumor in the oral cavity, is a disease with high recurrence and mortality rates. Butyrate, a metabolite produced by periodontal pathogens, has been linked to oral diseases. The purpose of this study was to evaluate the effect of sodium butyrate (NaB) on the proliferation, migration, and invasion of OSCC cells in vitro and to explore the potential mechanism.

Methods: Two OSCC cell lines (HSC-4 and SCC-9) were treated with NaB at different concentrations. The cell proliferation was assayed by CCK-8, ethylene deoxyuridine (EdU), and flow cytometry. Wound healing and transwell assay were performed to detect cell migration and invasion. Changes in epithelial–mesenchymal transition (EMT) markers, including E-cadherin, Vimentin, and SNAI1, were evaluated by quantitative real-time PCR (qRT-PCR), western blot, and immunofluorescent staining. The expression levels of matrix metalloproteinases (MMPs) were analyzed by qRT-PCR and ...

This document certifies that the manuscript listed above was copy edited for English language by LetPub, with regard to grammar, punctuation, spelling, and clarity. All of our language editors are native English speakers with long-term experience in editing scientific and technical manuscripts. We are committed to leveling the playing field for researchers whose native language is not English.

- Documents receiving this certification should be regarded as having undergone professional editorial revision for English language before submission. However, the authors may accept or reject LetPub's suggestions and changes at their own discretion and LetPub does not have editorial control over the submitted documents.
- The language quality of the submitted document is the sole responsibility of the submitting authors subject to those authors' adherence to LetPub's revisions and instruction. LetPub's provision of service does not constitute a guarantee or endorsement of the authors' work herein.
- Neither the research content nor the authors' intended meaning were altered in any way during the editing process.
- If you have any questions or concerns about this edited document, please contact us at support@letpub.com

LetPub
Your Manuscript Editor

LetPub is an author service brand owned and operated by Accdon LLC. Headquartered in the Boston area, we are a full-spectrum author services company with a large team of US-based certified language and scientific editors, ISO 17001 accredited translators, and professional scientific illustrators and animators. We advocate ethical publication practices and are an official member of the Committee on Publication Ethics (COPE).

For more information about our company, services, and partnership programs, please visit www.letpub.com.

© 2022 Accdon, LLC. All Rights Reserved. Tel: 1-781-202-9968 Email: info@accdon.com Address: 400 Fifth Ave, Suite 530, Waltham, MA 02451, United States
