# Supplementary figures and images for: Butyrate promotes oral squamous cell carcinoma cells migration, invasion and epithelial-mesenchymal transition
Source: PeerJ. 2022 Feb 22;10:e12991. doi: 10.7717/peerj.12991 (PMC8877342; doi:10.7717/peerj.12991)

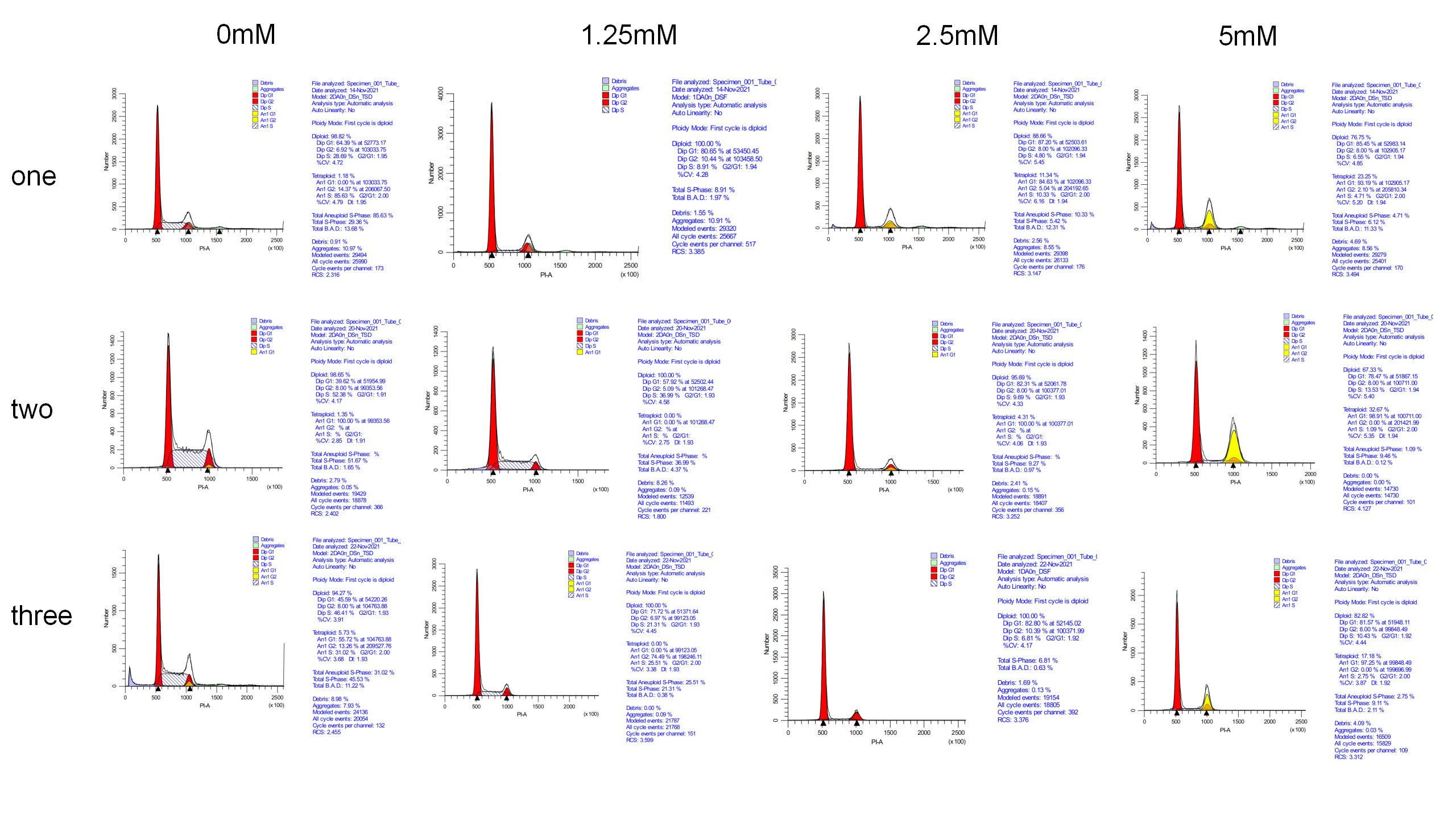

Supplement: Supplemental Information 1 [file peerj-10-12991-s001.zip › Raw data/Cell cycle analysis/HSC-4.png]

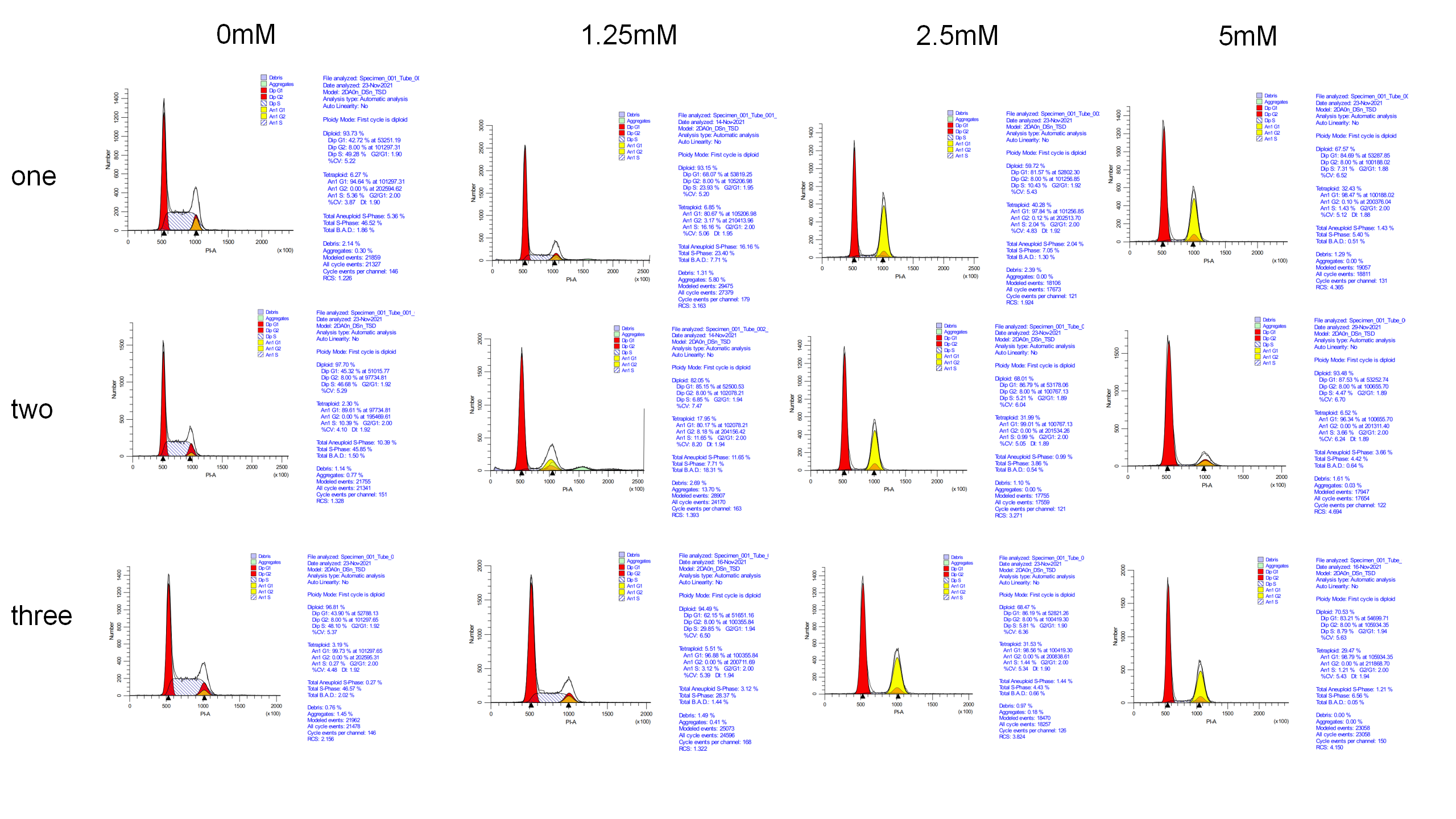

Supplement: Supplemental Information 1 [file peerj-10-12991-s001.zip › Raw data/Cell cycle analysis/SCC-9.png]

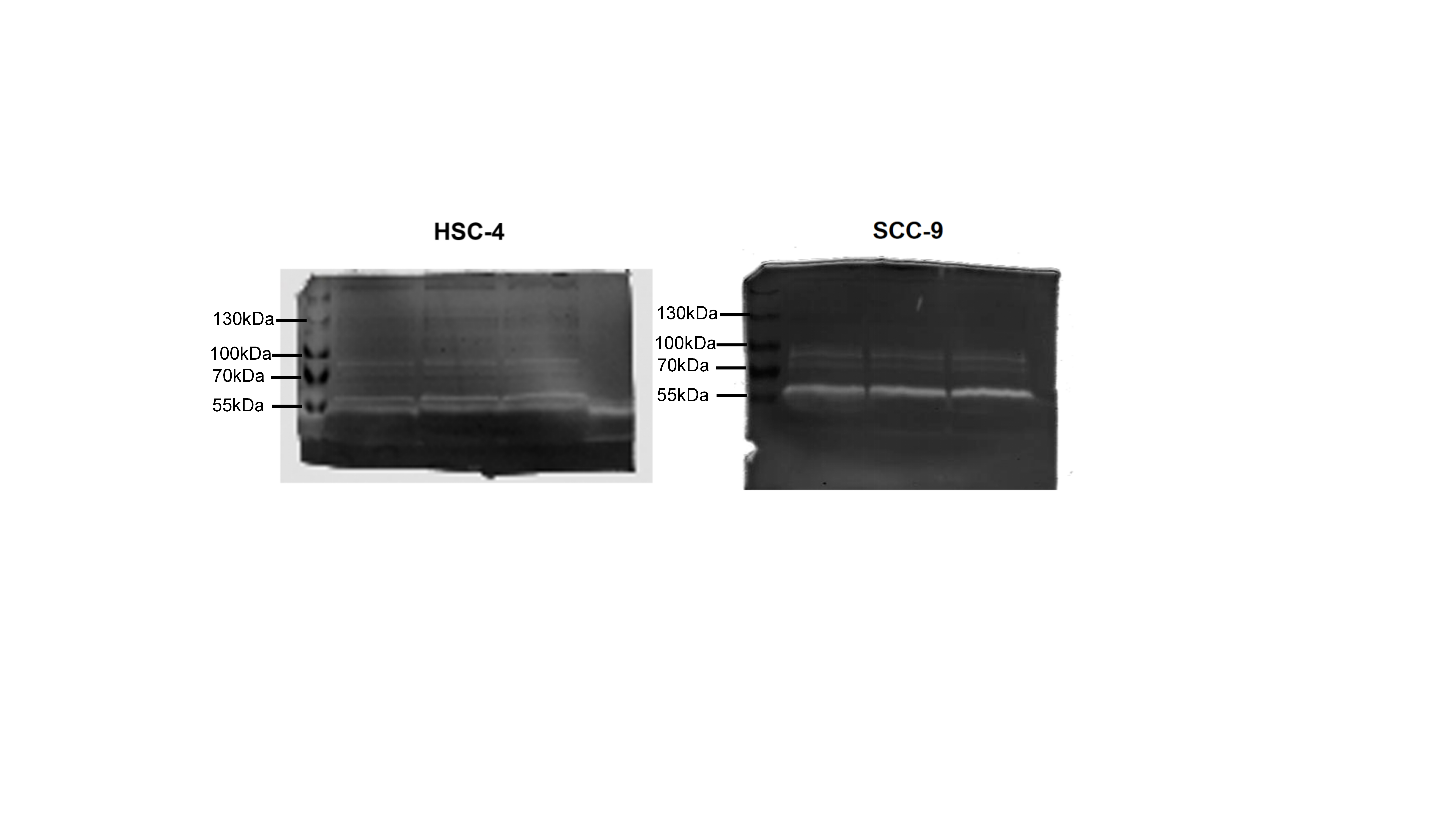

Supplement: Supplemental Information 1 [file peerj-10-12991-s001.zip › Raw data/Gelatin zymography/HSC-4 and SCC-9.png]

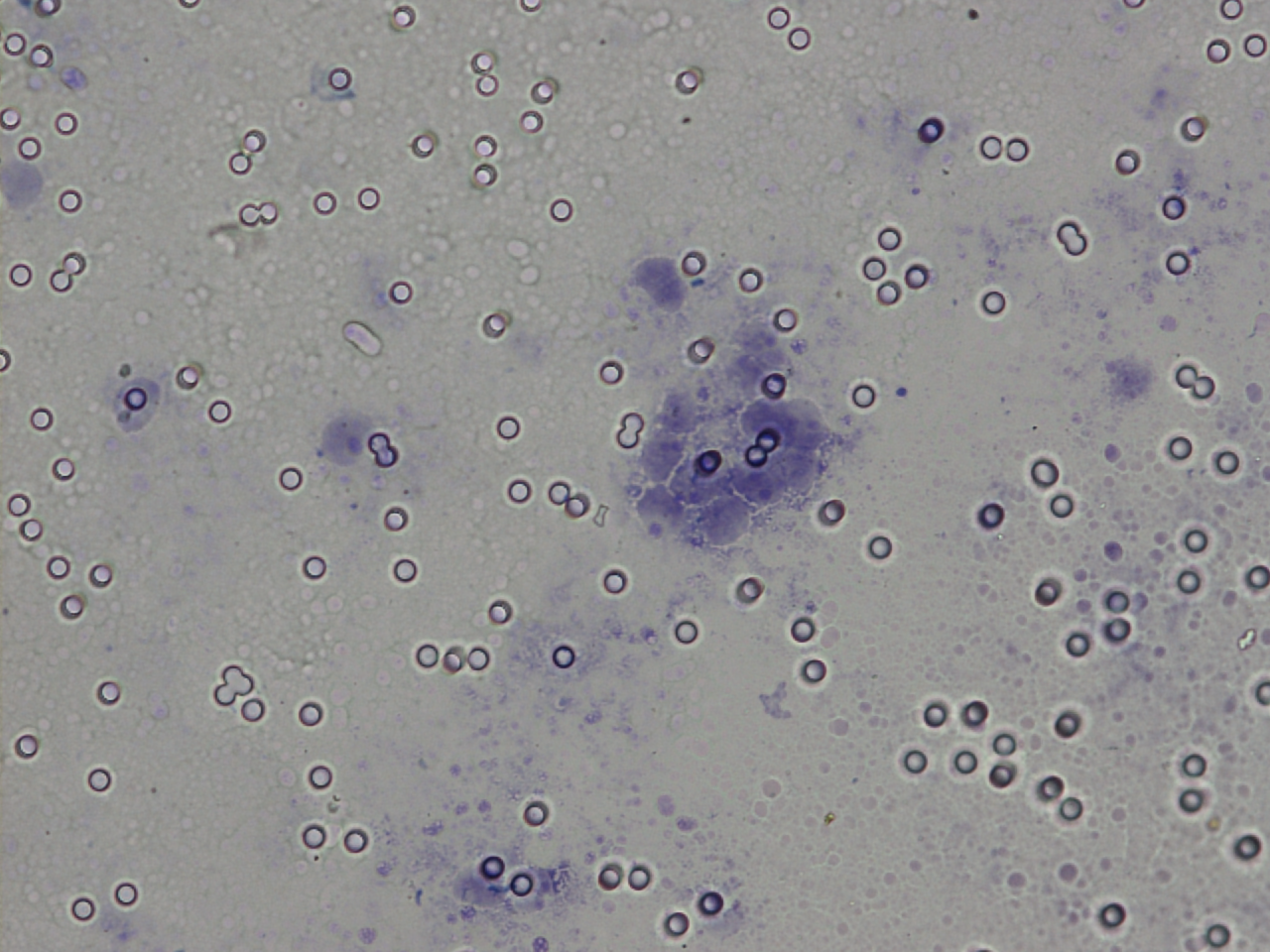

Supplement: Supplemental Information 1 [file peerj-10-12991-s001.zip › Raw data/Transwell/invasion/HSC-4-0mM.png]

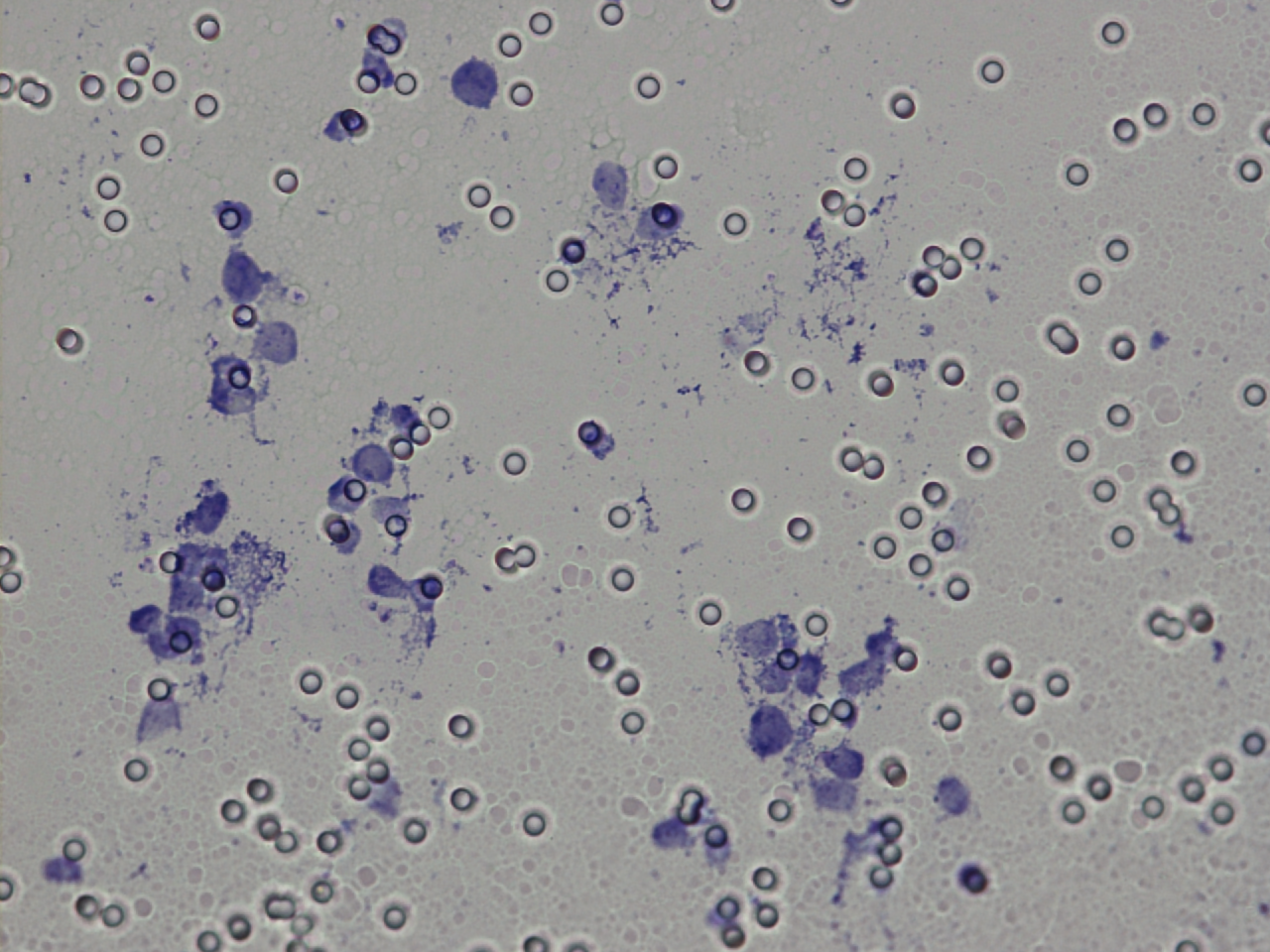

Supplement: Supplemental Information 1 [file peerj-10-12991-s001.zip › Raw data/Transwell/invasion/HSC-4-2.5mM.png]

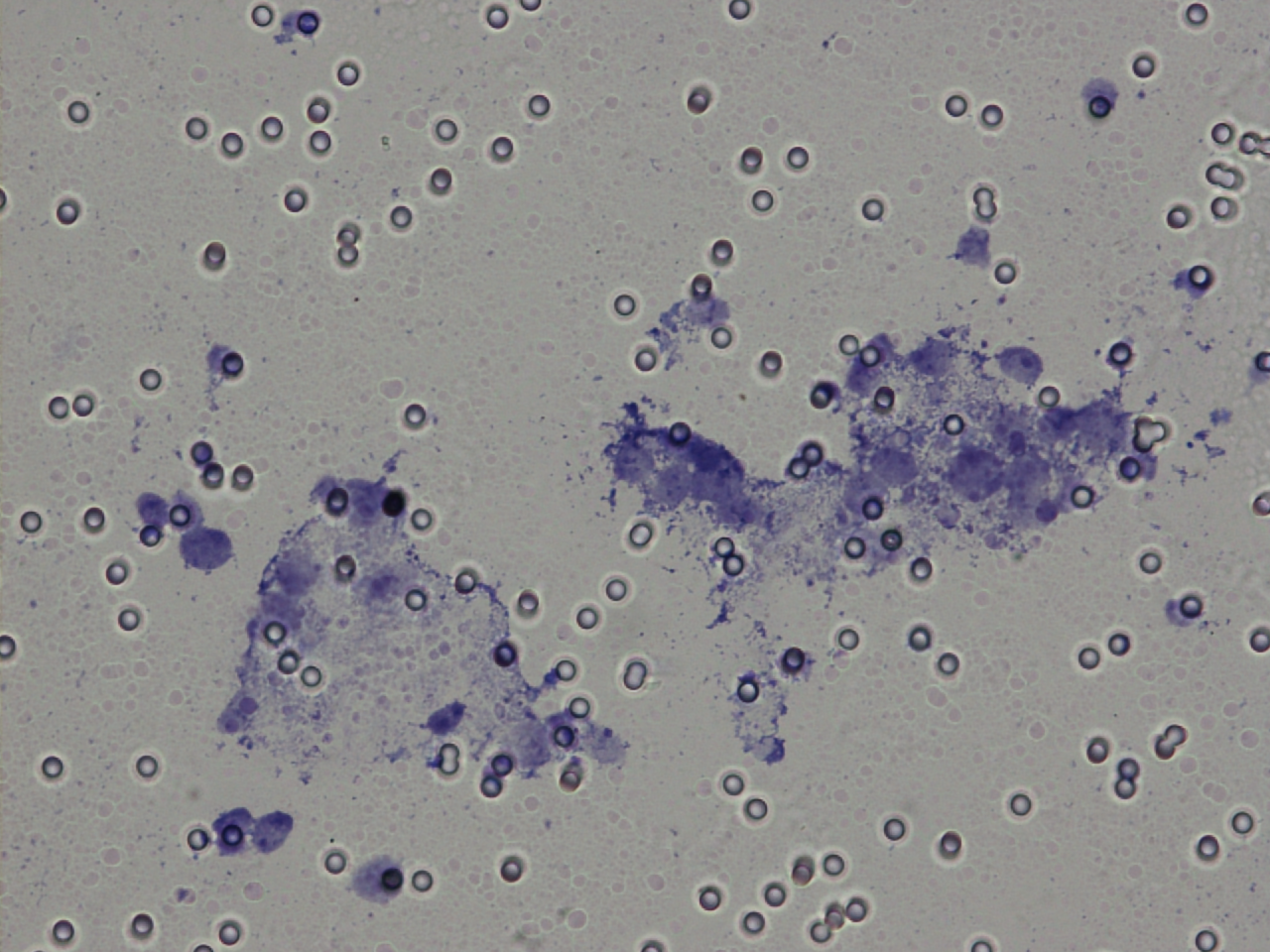

Supplement: Supplemental Information 1 [file peerj-10-12991-s001.zip › Raw data/Transwell/invasion/HSC-4-5mM.png]

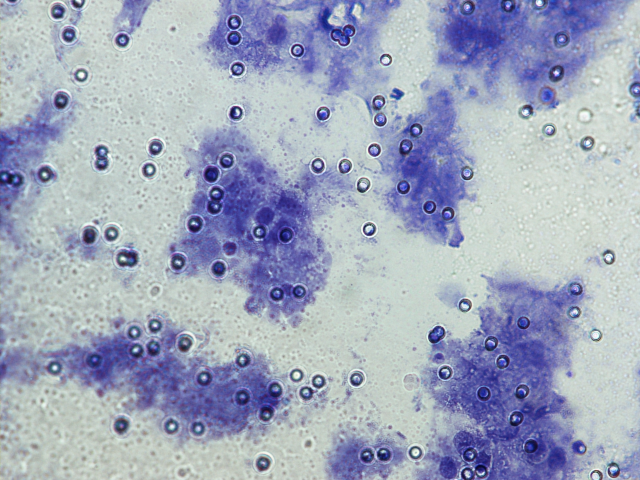

Supplement: Supplemental Information 1 [file peerj-10-12991-s001.zip › Raw data/Transwell/invasion/SCC-9-0Mm.png]

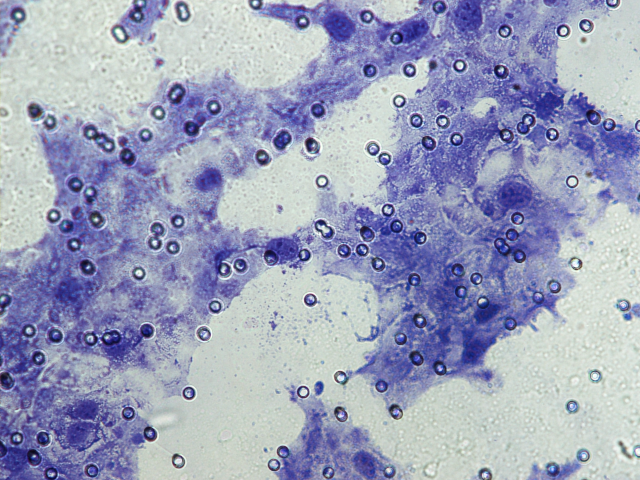

Supplement: Supplemental Information 1 [file peerj-10-12991-s001.zip › Raw data/Transwell/invasion/SCC-9-2.5mM.png]

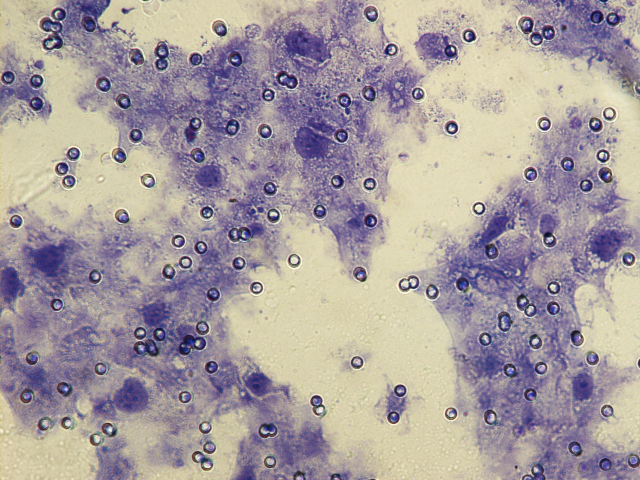

Supplement: Supplemental Information 1 [file peerj-10-12991-s001.zip › Raw data/Transwell/invasion/SCC-9-5mM.png]

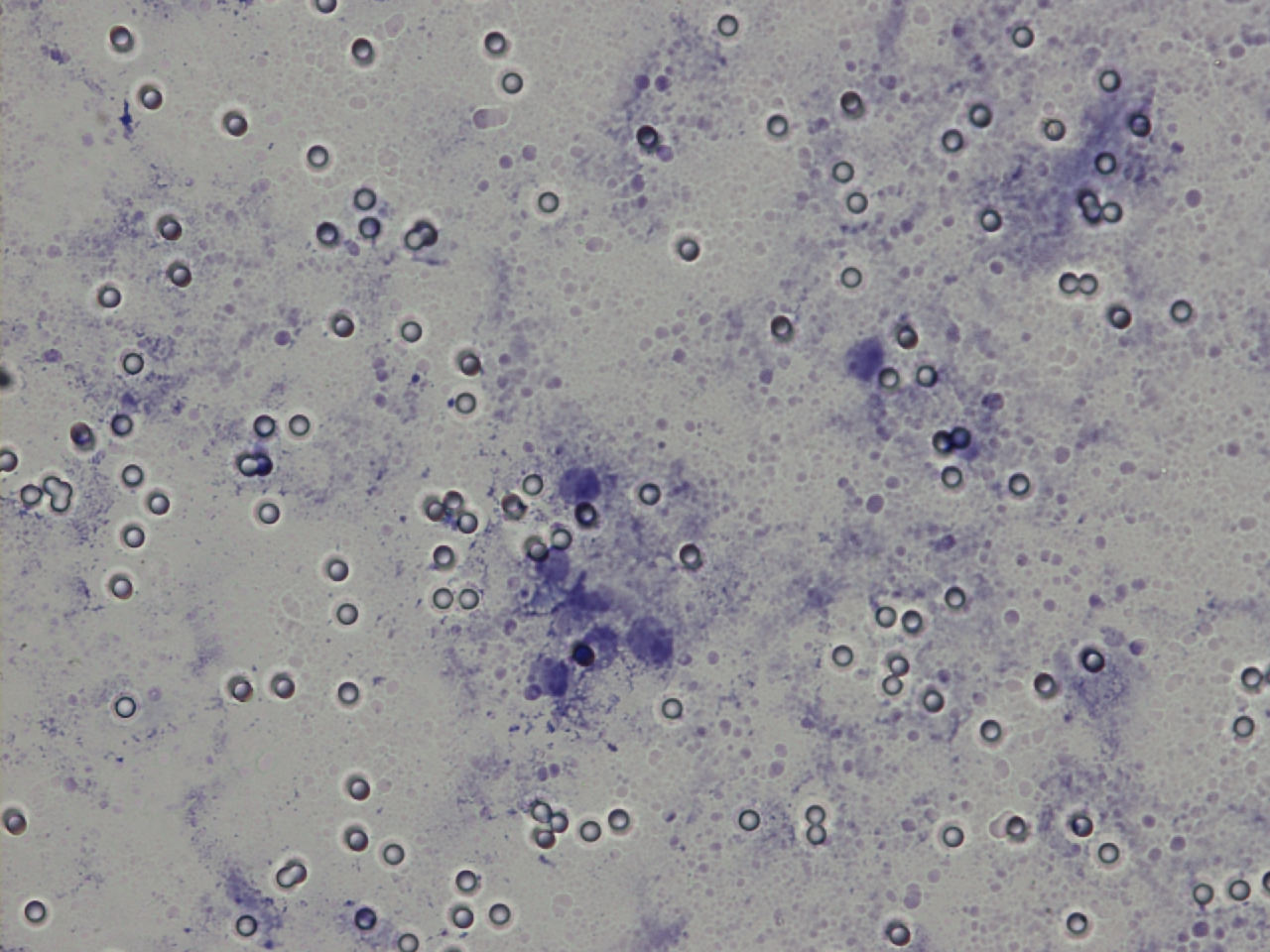

Supplement: Supplemental Information 1 [file peerj-10-12991-s001.zip › Raw data/Transwell/migration/HSC-4-0mM.png]

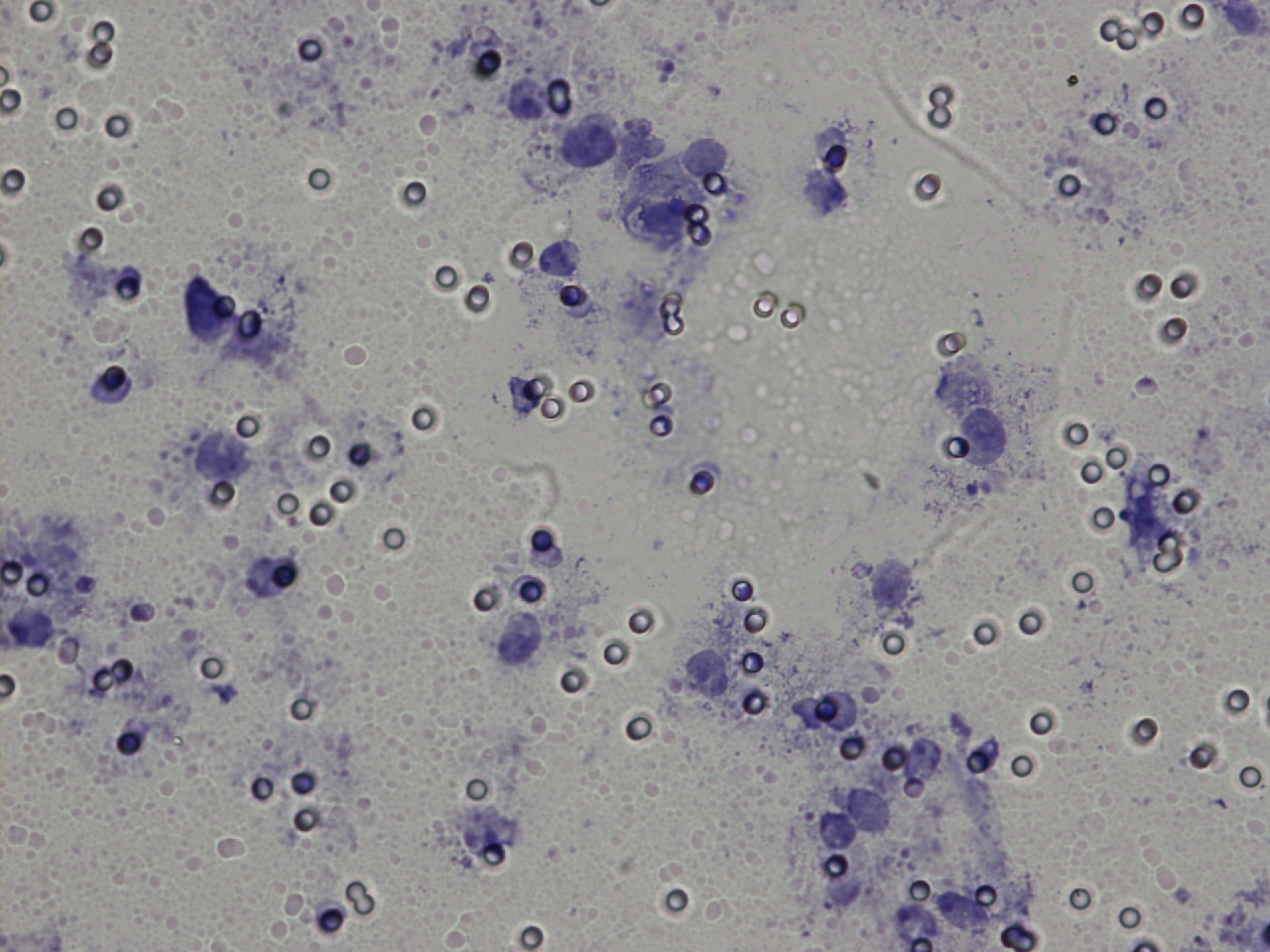

Supplement: Supplemental Information 1 [file peerj-10-12991-s001.zip › Raw data/Transwell/migration/HSC-4-2.5mM.png]

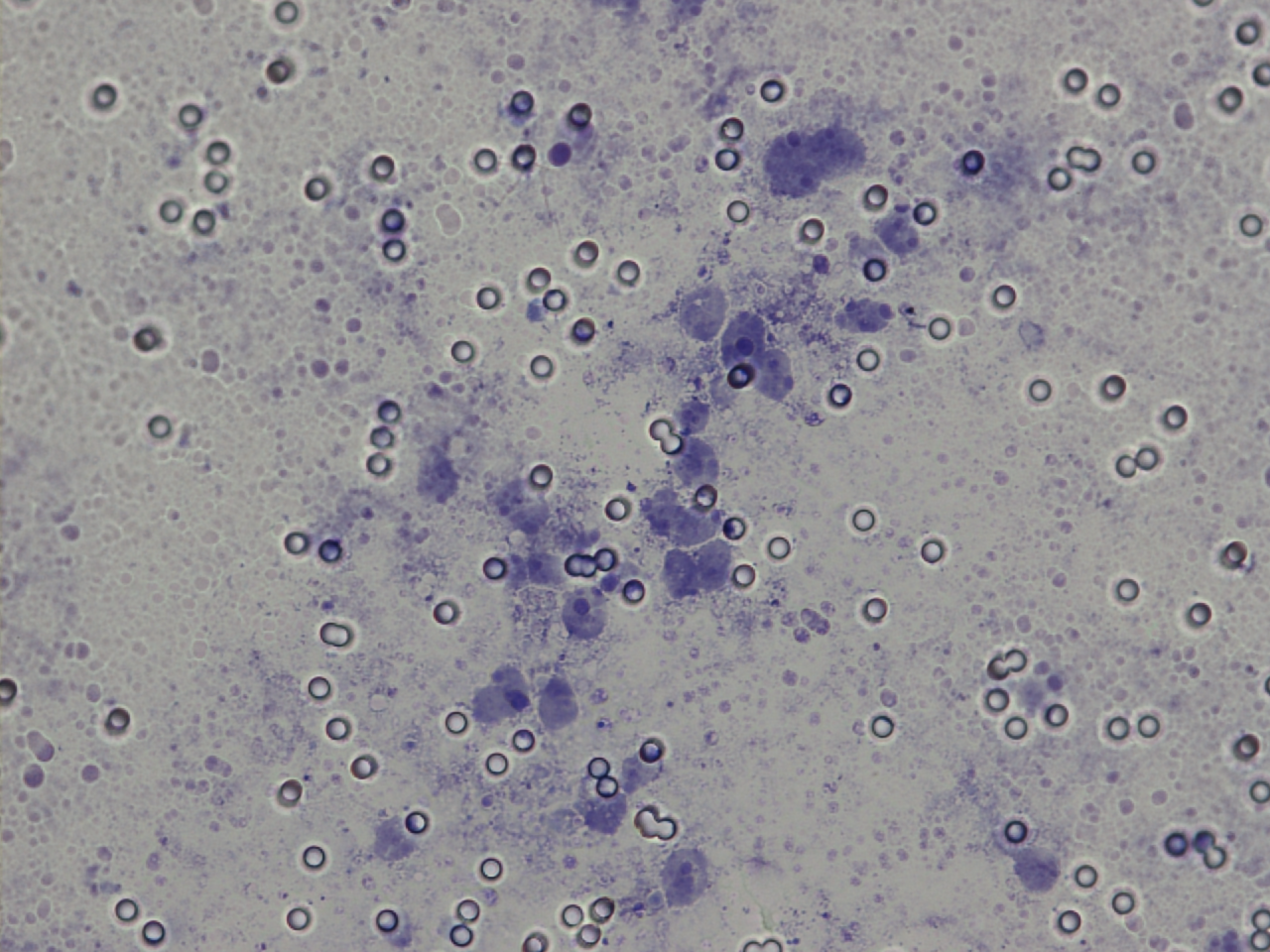

Supplement: Supplemental Information 1 [file peerj-10-12991-s001.zip › Raw data/Transwell/migration/HSC-4-5mM.png]

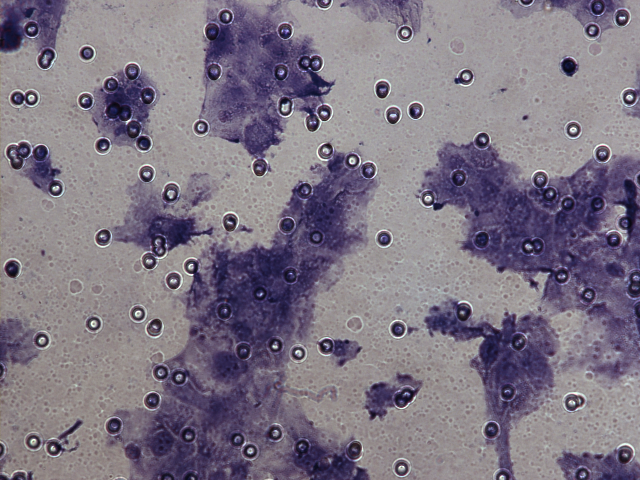

Supplement: Supplemental Information 1 [file peerj-10-12991-s001.zip › Raw data/Transwell/migration/SCC-9-0mM.png]

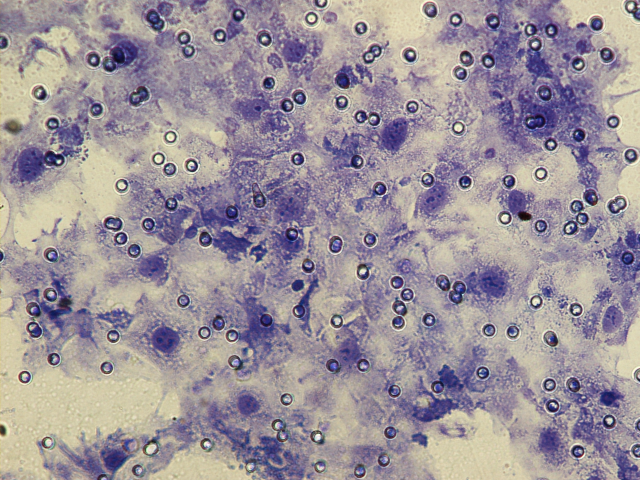

Supplement: Supplemental Information 1 [file peerj-10-12991-s001.zip › Raw data/Transwell/migration/SCC-9-2.5mM.png]

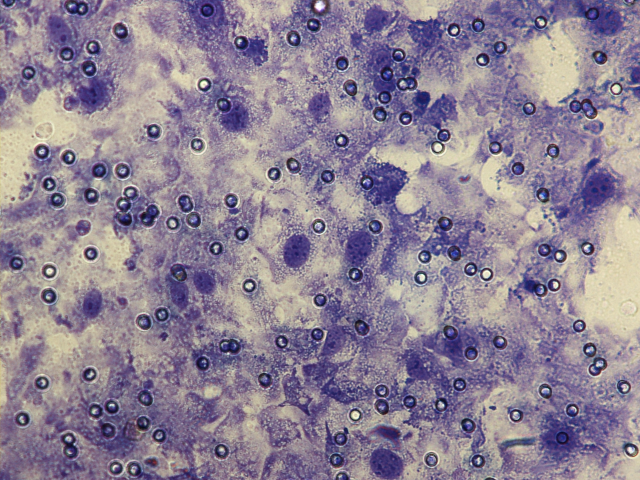

Supplement: Supplemental Information 1 [file peerj-10-12991-s001.zip › Raw data/Transwell/migration/SCC-9-5mM.png]

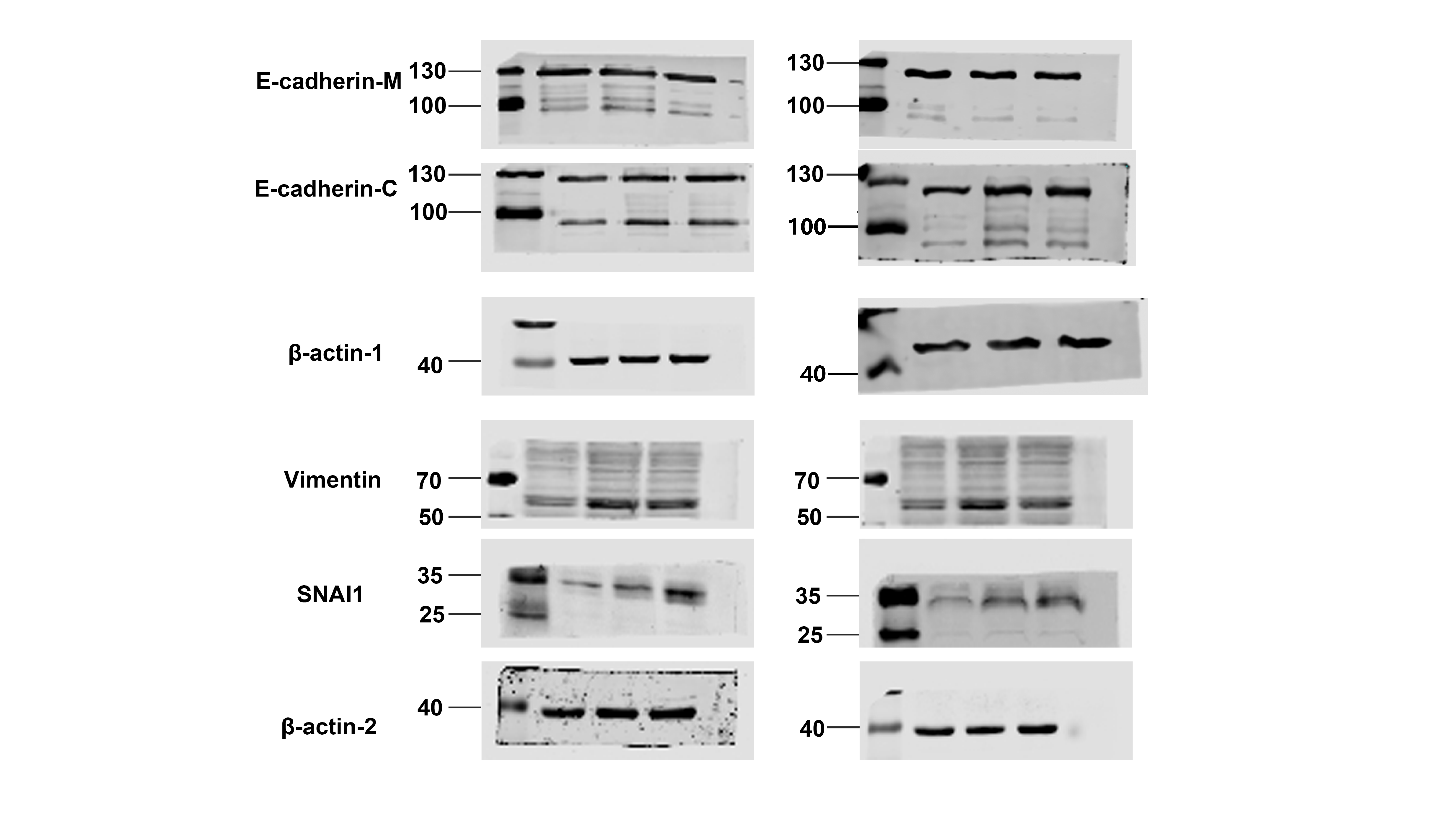

Supplement: Supplemental Information 1 [file peerj-10-12991-s001.zip › Raw data/Western blot/western blot.png]

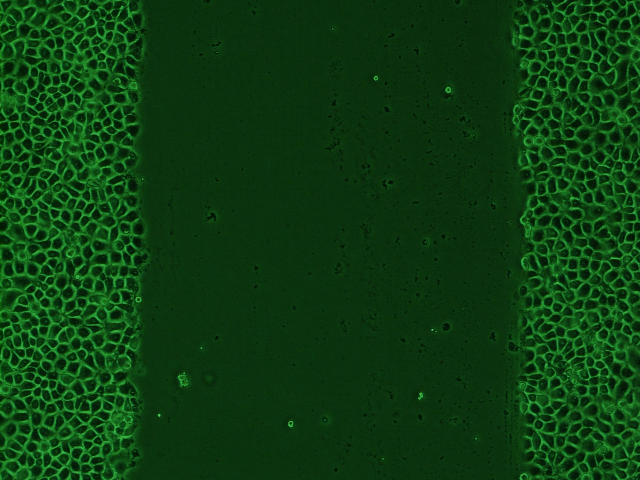

Supplement: Supplemental Information 1 [file peerj-10-12991-s001.zip › Raw data/Wound healing/HSC-4/0-0h.tif]

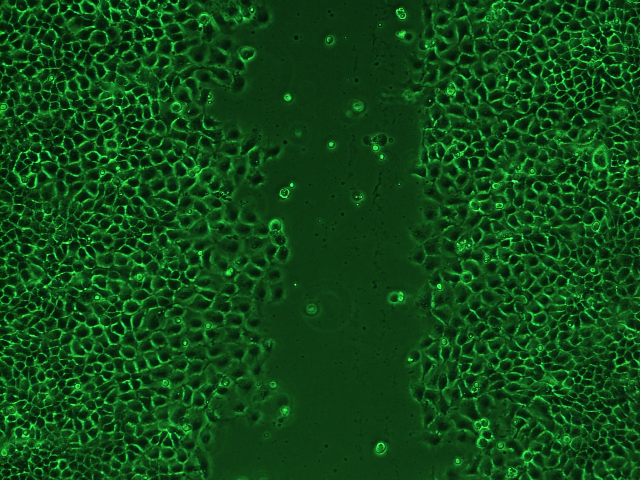

Supplement: Supplemental Information 1 [file peerj-10-12991-s001.zip › Raw data/Wound healing/HSC-4/0-24h.tif]

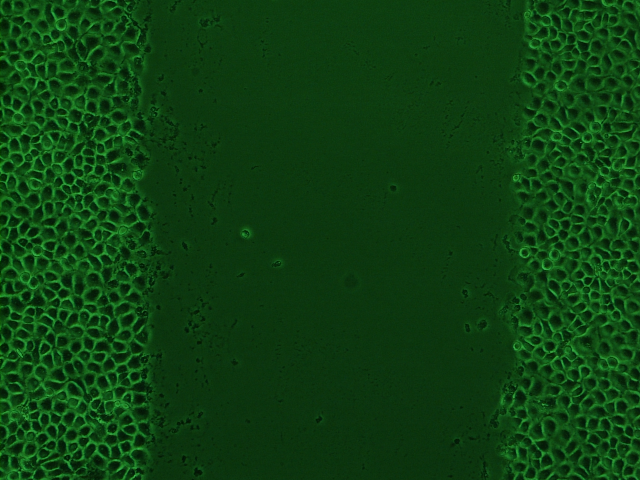

Supplement: Supplemental Information 1 [file peerj-10-12991-s001.zip › Raw data/Wound healing/HSC-4/2.5-0h.tif]

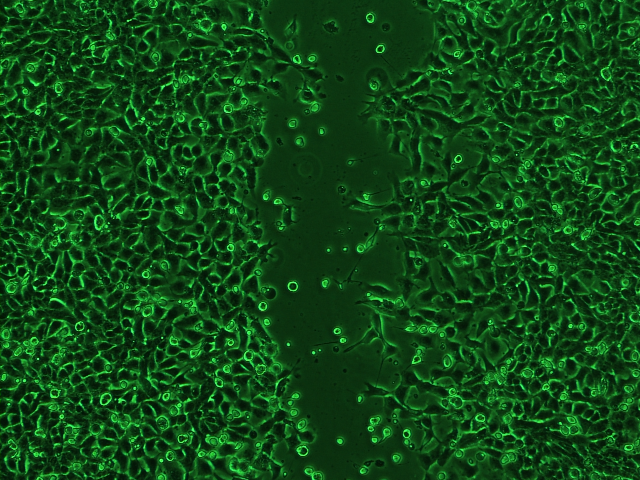

Supplement: Supplemental Information 1 [file peerj-10-12991-s001.zip › Raw data/Wound healing/HSC-4/2.5-24h.tif]

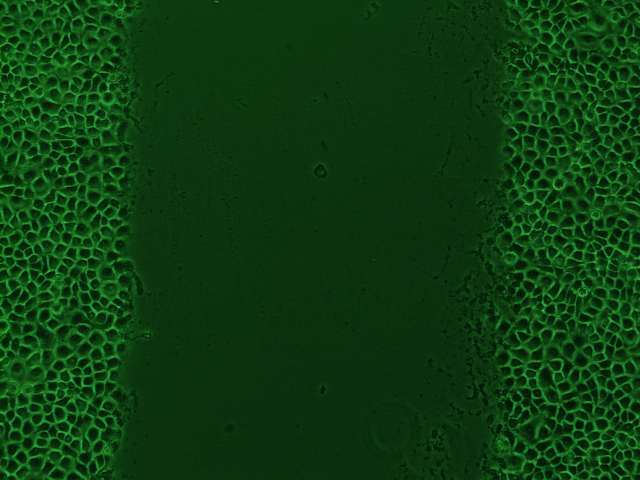

Supplement: Supplemental Information 1 [file peerj-10-12991-s001.zip › Raw data/Wound healing/HSC-4/5-0h.tif]

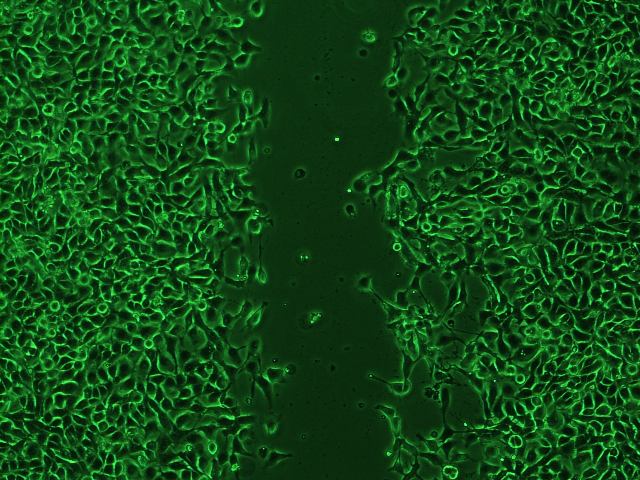

Supplement: Supplemental Information 1 [file peerj-10-12991-s001.zip › Raw data/Wound healing/HSC-4/5-24h.tif]

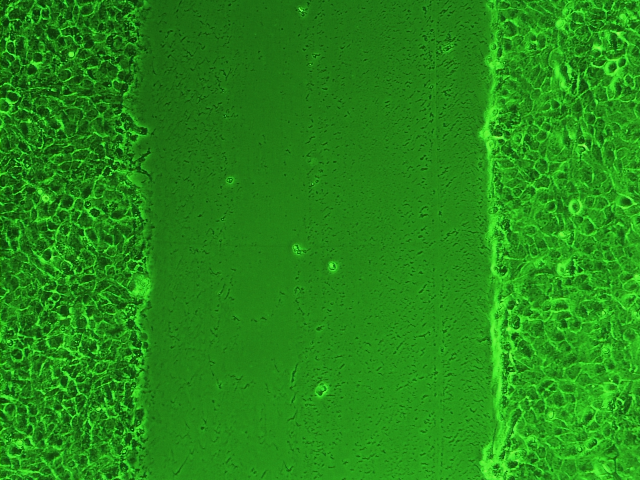

Supplement: Supplemental Information 1 [file peerj-10-12991-s001.zip › Raw data/Wound healing/SCC-9/0-0h.tif]

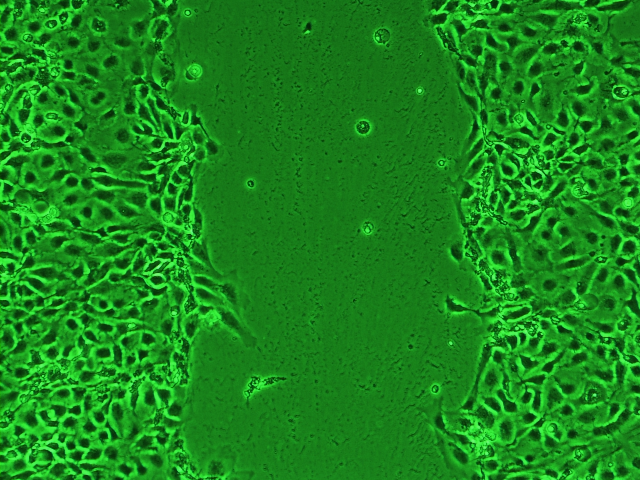

Supplement: Supplemental Information 1 [file peerj-10-12991-s001.zip › Raw data/Wound healing/SCC-9/0-24h.tif]

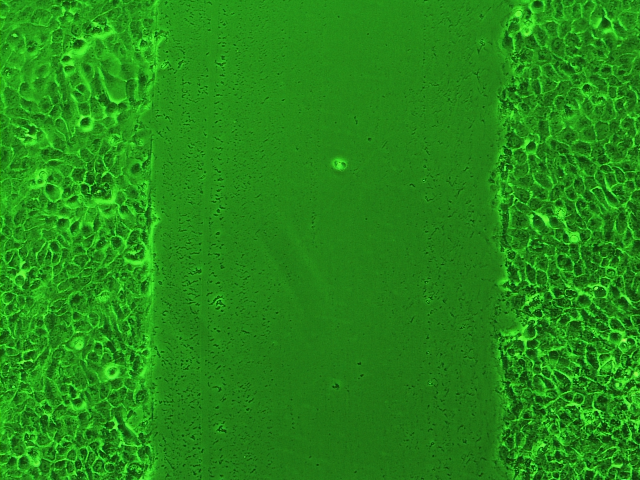

Supplement: Supplemental Information 1 [file peerj-10-12991-s001.zip › Raw data/Wound healing/SCC-9/2.5-0h.tif]

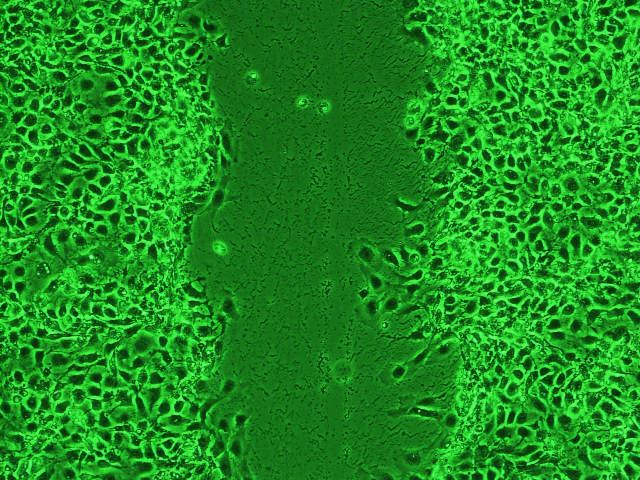

Supplement: Supplemental Information 1 [file peerj-10-12991-s001.zip › Raw data/Wound healing/SCC-9/2.5-24h.tif]

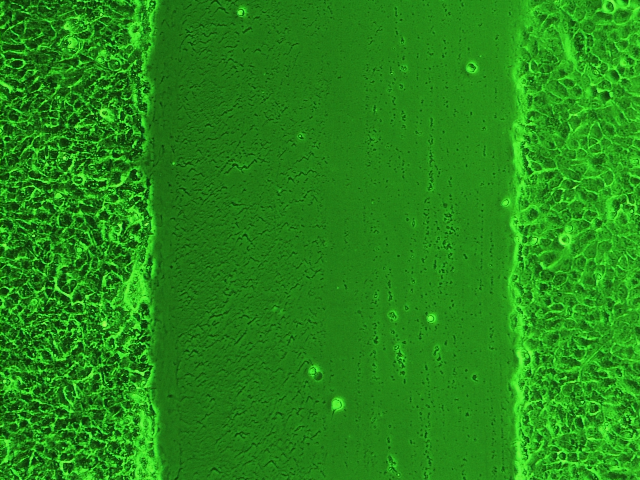

Supplement: Supplemental Information 1 [file peerj-10-12991-s001.zip › Raw data/Wound healing/SCC-9/5-0h.tif]

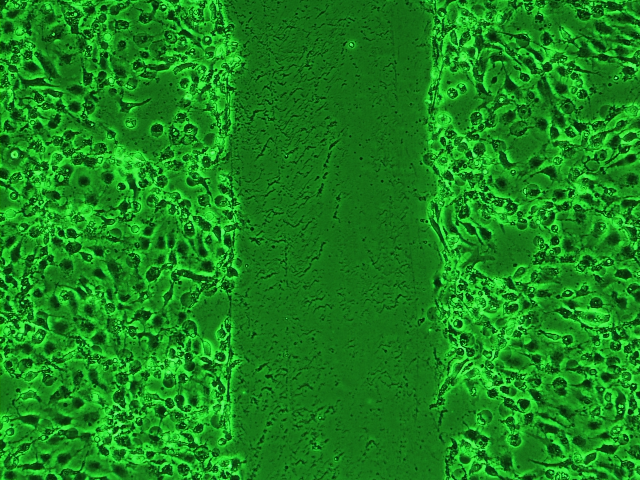

Supplement: Supplemental Information 1 [file peerj-10-12991-s001.zip › Raw data/Wound healing/SCC-9/5-24h.tif]
