# Supplementary material for: Butyrate promotes oral squamous cell carcinoma cells migration, invasion and epithelial-mesenchymal transition
Source: PeerJ. 2022 Feb 22;10:e12991. doi: 10.7717/peerj.12991 (PMC8877342; doi:10.7717/peerj.12991)
Supplement: Supplemental Information 2 [file peerj-10-12991-s002.docx]

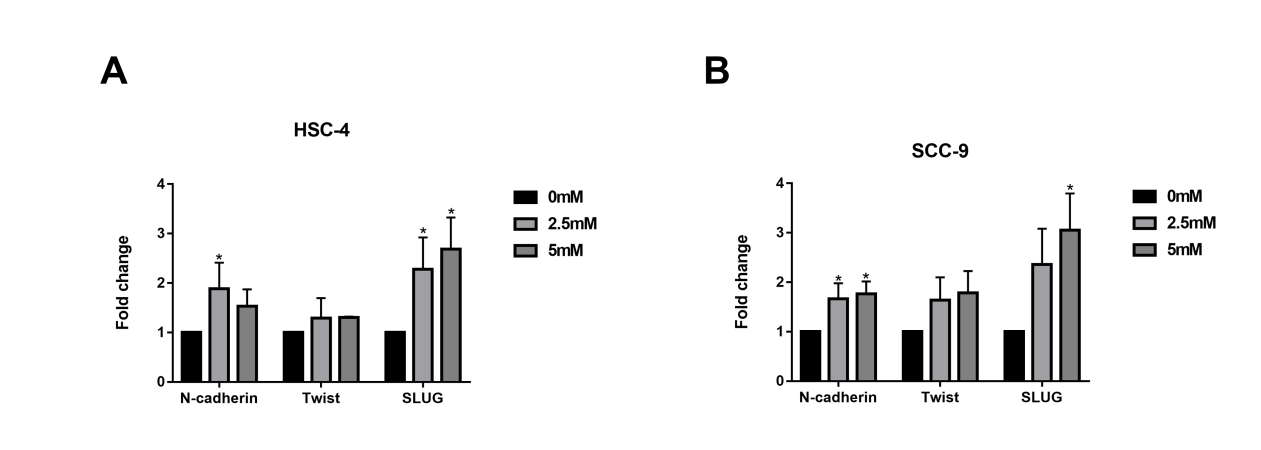


(A, B) qRT-PCR analysis was used to detected the gene expression of N-cadherin, Twist and SLUG in HSC-4 and SCC-9 cells after treatment with NaB for 24h. Data are presented as the mean ± SD of at least three independent experiments. **P* < 0.05 vs. control (NaB, 0 mM).
